# Trauma-induced regulation of VHP-1 modulates the cellular response to mechanical stress

**DOI:** 10.1038/s41467-021-21611-8

**Published:** 2021-03-05

**Authors:** Nathan Egge, Sonja L. B. Arneaud, Rene Solano Fonseca, Kielen R. Zuurbier, Jacob McClendon, Peter M. Douglas

**Affiliations:** 1 Department of Molecular Biology, UT Southwestern Medical Center, Dallas, TX USA; 2 Medical Scientist Training Program, UT Southwestern Medical Center, Dallas, TX USA; 3 Hamon Center for Regenerative Science and Medicine, UT Southwestern Medical Center, Dallas, TX USA

**Keywords:** Cell biology, Genetics, Molecular biology

## Abstract

Mechanical stimuli initiate adaptive signal transduction pathways, yet exceeding the cellular capacity to withstand physical stress results in death. The molecular mechanisms underlying trauma-induced degeneration remain unclear. In the nematode *C. elegans*, we have developed a method to study cellular degeneration in response to mechanical stress caused by blunt force trauma. Herein, we report that physical injury activates the c-Jun kinase, KGB-1, which modulates response elements through the AP-1 transcriptional complex. Among these, we have identified a dual-specificity MAPK phosphatase, VHP-1, as a stress-inducible modulator of neurodegeneration. VHP-1 regulates the transcriptional response to mechanical stress and is itself attenuated by KGB-1-mediated inactivation of a deubiquitinase, MATH-33, and proteasomal degradation. Together, we describe an uncharacterized stress response pathway in *C. elegans* and identify transcriptional and post-translational components comprising a feedback loop on Jun kinase and phosphatase activity.

## Abstract (web summary)

The nervous system utilizes adaptive strategies to mitigate trauma-induced neurodegeneration. Here, the authors report that a MAPK phosphatase VHP-1 is induced by trauma and regulates transcriptional responses to mechanical stress in a *C. elegans* model.

## Introduction

Organisms are constantly at odds with harsh environmental or physiological stimuli. Cells must be able to sense and mount a response to these stimuli in order to adapt and maintain homeostasis. Mechanical forces impose tensile, compressive, or shear strain on cells and tissues that can alter the shape, size, organelle arrangement, and/or chromatin structure^1,2^. Mechanotransduction, or the conversion of mechanical forces into chemical and electrical signals, enables cellular responsiveness to physical stimuli. These include activation of several classes of protein kinases, small G-proteins, phospholipases, and direct signaling to the nucleus through the nesprin/SUN1 complex^3^. Dependent on cellular physiology and the magnitude of force, stimuli can promote proliferation, differentiation, or beneficial protein translation, as in the case for chondrocytes and select tumor cells^4,5^. Conversely, harsh stimuli can promote cell death or chronic dysfunction, as observed upon hypertension, tissue laceration, or blunt force trauma to the brain, kidney, liver, or muscle^6–10^.

While primary mechanisms for direct mechanosensation have been described in every domain of life^11^, less clear are the downstream molecular events utilized by cells to respond to harsh mechanical stimuli and abrogate long-term deleterious effects^12^. To date, perhaps the best understood molecular response to mechanical stressors has been described in the heart. Cardiomyocytes experience increased mechanical load due to hypertension or inherent genetic defects^13^. Excessive load may lead to apoptosis or cardiac tissue dysfunction and subsequent pathological hypertrophy. At the molecular level, activation of integrins^14,15^, G protein-coupled receptors^16^, and mechanosensitive cation channels^17^ represent major mechanisms of mechanosensation. These signaling events operate through several downstream pathways including calcium influx^18^, protein kinase C (PKC)^19^, mitogen-activated protein kinases (MAPKs), the Janus kinase/signal transducer and activator of transcription pathway (JAK/STAT)^20^, focal adhesion kinase (FAK)^21^, and several small GTPases including Ras, RhoA, and Rac1^22,23^. Recent investigations into regenerative mechanisms following nerve transection or tissue laceration have likewise highlighted several downstream signaling factors. These model systems of harsh stimuli involve immediate intercellular dissociation and plasma membrane rupture. Similar to cardiomyocytes, roles for the JAK/STAT^24^ and MAPK pathways^25–29^ have been implicated in these models.

Distinct from chronic mechanical loading or direct tissue laceration, blunt force trauma involves the transient application of intense force that causes membrane deformation, organelle rearrangement, and disrupted intercellular interactions without necessarily compromising cellular or tissue integrity. The mechanical stress resulting from blunt force trauma can initiate progressive cellular degeneration and death, as observed in traumatic brain injury, blunt chest trauma, and trauma-induced rhabdomyolysis^7,30,31^. However, molecular events detailing the process from mechanical stress to the ensuing pathology are poorly understood, in part due to the lack of available model systems that are amenable to high-throughput study.

In this work, we examine the nematode *C. elegans* to understand how post-mitotic cells within an intact organism respond to mechanical stress caused by blunt force trauma. In particular, we develop and characterize a method to administer blunt force injury to large, synchronous populations of adult worms. With this trauma model, we define a distinct mechanical stress response and discover response elements critical for cell survival through candidate-based RNAi screening. From these studies, we identify the MAPK phosphatase, VHP-1, as a vital response element that negatively regulates its upstream Jun kinase, KGB-1, and itself is negatively regulated by stress-inactivation of a deubiquitinase, MATH-33. We propose that the regulation of VHP-1 represents a negative feedback loop by which transcriptional and post-translational mechanisms coordinate induction and attenuation of stress response signaling.

## Results

### Blunt force trauma elicits neuronal defects in *C. elegans*

**Fig. 1:**
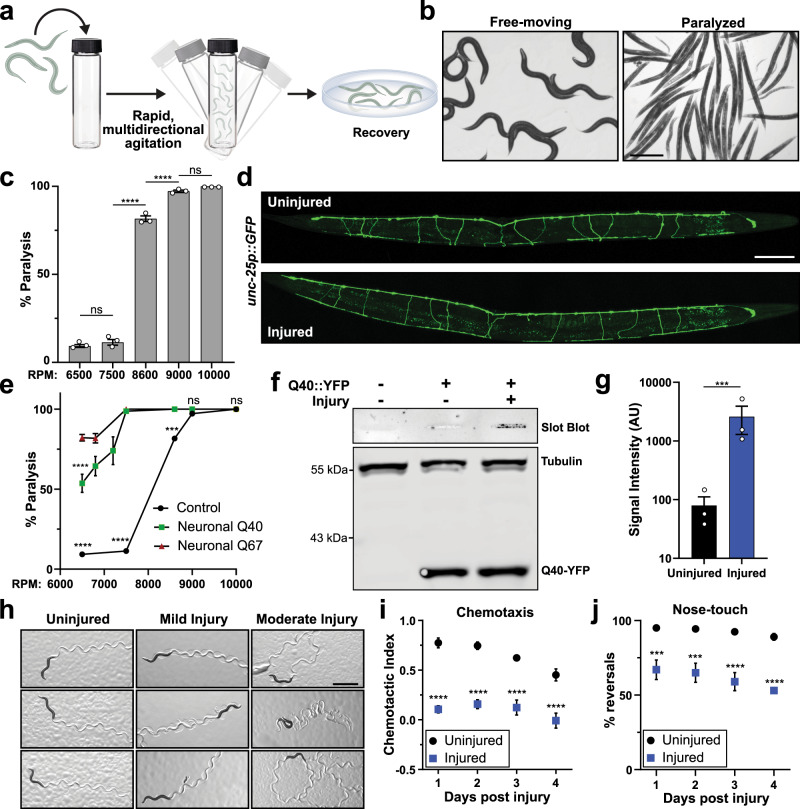
Blunt force trauma induces neuronal dysfunction in *C. elegans*. **a** Diagram depicting method of delivering blunt trauma to worms. **b** Micrographs of worms exhibiting movement with normal body bend (left) versus post injury paralysis (right). Reference Fig. 1c. Scale = 400 µm. **c** Percent of animals entering paralytic state immediately post injury is dependent on agitation frequency. Mean ± SEM, ns (not significant) *p* = 0.7044 and 0.4494, ****p < 0.0001 by two-way ANOVA with Tukey multiple comparison test. *n* = 3 independent trials. **d** Fluorescence maximum projections of Z-stacks taken by confocal microscopy of worms expressing GFP in GABAergic neurons. Worms imaged either uninjured or 1 h post injury. Reference Supplementary Fig. 1b. Scale = 100 µm. **e** Paralytic response to injury in either a control worm strain (from Fig. 1c, black circles), or worms expressing polyglutamine expansions Q40::YFP (soluble, green squares) or Q67::YFP (insoluble, red triangles) in neurons. Mean ± SEM, ****p < 0.0001, ***p = 0.0001 by two-way ANOVA with Tukey test, *n* = 3 independent trials. **f** Filter trap analysis of SDS-resistant aggregates on cellulose acetate membranes from neuronally expressed Q40::YFP worms either uninjured or 48 h post injury. Western blots for the respective proteins resolved by SDS-PAGE in the bottom panel. **g** Quantification of filter trap. Shown are log-transformed values of slot blot band intensity with black and blue denoting uninjured and injured worms, respectively (corrected for loading by YFP signal). Mean ± SEM, **p = 0.0008 by two-sided ratio-paired *t*-test, *n* = 3 biological replicates. **h** Micrographs of worm tracts on *E. coli* lawn to assess movement 48 h after receiving no injury, non-paralytic mild injury (7500 rpm), or paralytic moderate injury (8600 rpm). Scale = 1 mm. **i** Chemotaxis of uninjured (black circles) or injured worms (blue squares) with butanol 24–96 h post injury. Chemotactic index of 1.0 indicates 100% preference for butanol and 0.0 indicates no preference. Mean ± SEM, ****p < 0.0001 and uninjured Day 1 vs Day 4 ***p = 0.0005 by two-way ANOVA with Tukey test, *n* = 6 independent trials for day 1, 2, 4 and *n* = 3 independent trials for day 3. **j** Gentle nose touch assay of uninjured (black circles) or injured worms (blue squares), in which a stop and reversal of worm movement indicated a positive response. Mean ± SEM, ***p = 0.0006 and 0.0004, ****p < 0.0001 when compared to uninjured by two-way ANOVA with Tukey test, *n* = 200 animals per condition over three independent trials.

We have developed a multi-impact model of blunt force trauma in *C. elegans* utilizing high frequency, multidirectional agitation, which simultaneously delivers a rapid acceleration/deceleration injury to a large population of age-synchronized animals (Fig. 1a). Adjusting the magnitude of the mechanical injuries resulted in dose-dependent animal paralysis (Fig. 1b,c). By adjusting agitation frequency, we have designated three levels of injury, namely mild, moderate, and severe. Agitation frequencies associated with mild injury failed to induce animal paralysis, whereas moderate injury at 8600 rpm induced a non-lethal state of temporary paralysis in 82% of the population (Fig. 1c). Severe injury at 10,000 rpm resulted in prolonged and, in some cases, irreversible paralysis or even death in 49% of the population (Supplementary Fig. 1a). To determine if the paralytic injury was associated with direct transection of neuronal processes or lysis of cell bodies, we examined worms expressing GFP in GABAergic neurons as performed in nerve transection studies^32,33^. Confocal microscopy revealed few irregularities in neuronal commissures throughout the body immediately after injury (Fig. 1d, Supplementary Fig. 1b). Likewise, injured worms did not display dye-filling defects when stained with the lipophilic neuron-labeling dye, DiI (Supplementary Fig. 1c, d), indicating that neuronal processes in the nose tip were not immediately compromised by trauma^34^. Worms expressing LifeAct::mRuby in their body-wall muscles^35^, which specifically labels the sarcomeric actin filaments, revealed that muscular architecture remained unperturbed in the majority (77%) of worms imaged by confocal microscopy (Supplementary Fig. 1e–g). In addition, transmission electron microscopy (TEM) of injured worms revealed normal sarcomeric structures in muscle cells (Supplementary Fig. 1h).

We were interested if the observed paralytic phenotype upon injury was in part due to immediate neuronal dysfunction in the absence of neuronal lysis. To this end, we hypothesized that disrupting normal neurological activity through impairment of protein homeostasis would hypersensitize worms to trauma-induced paralysis. Ectopic expression of unstable, disease-linked proteins disrupts protein homeostasis and promotes neuronal dysfunction^36^. Previously shown to impair cellular function^37^, the pan-neuronal expression of either a soluble (Q40) or insoluble (Q67) YFP-tagged poly-glutamine expansion protein was sufficient to sensitize worms to paralysis (Fig. 1e). Furthermore, trauma initiated SDS-resistant aggregation of the normally soluble Q40::YFP in neurons (Fig. 1f, g). These data suggest a reciprocal relationship between neuronal proteostasis and mechanical stress.

The majority of paralyzed worms recovered movement within 2–8 h of moderate injury providing further support that trauma did not elicit gross morphologic damage. Within 48 h of injury, 90% of injured worms were mobile (Supplementary Fig. 1i). Despite recovery from paralysis, both mild and moderate trauma generated abnormal movement phenotypes (Fig. 1h, Supplementary Fig. 1i). Locomotion in non-injured *C. elegans* primarily consisted of a regular sinusoidal pattern of set amplitude in 74% of worms, as observed by the typical *C. elegans* locomotion pattern on a bacterial lawn (Fig. 1h, Supplementary Fig. 1i). Sub-paralytic mild trauma resulted in 47% of worms exhibiting abnormal movement, which predominately fell into two phenotypes: increased sinusoidal amplitude and increased path curvature (Supplementary Fig. 1i). Paralytic moderate injury further exacerbated movement defects in 86% of worms, which were classified into one of five main categories: increased sinusoidal amplitude (19% of worms), decreased sinusoidal amplitude (16%), increased curvature (23%), erratic movement (12%), and no movement (10%). The remainder of the current study focuses on moderate injury conditions.

The frequency of abnormal movement post trauma eclipsed the proportion of worms exhibiting gross muscular or neurological signs of damage. We hypothesized that neurological dysfunction, rather than gross tissue disruption, contributed to the observed phenotypes. To determine if locomotion defects coincided with the development of other neurologic deficiencies, we assessed how trauma affected both chemo- and mechano-sensation. Injured worms displayed impaired chemotaxis, showing significantly decreased preference for chemoattractants post injury (Fig. 1i), indicating defects in the AWA, AWB, and/or AWC chemosensory neurons or the post-synaptic AIB, AIY, and AIZ interneurons^38^. Furthermore, injured animals exhibited an age-dependent decline in gentle nose-touch mechanosensation (Fig. 1j), indicating defective signaling through the ASH, FLP, OLQ, or IL1 neurons^38^. Collectively, the progressive coordination and sensory defects may not stem from immediate disruption of neuronal integrity, as in axotomy models, or in immediate gross muscular damage. Instead, these data suggest mechanical trauma results in non-lytic, axon-sparing neuronal injury that prompts immediate and long-term neuronal sensory, motor, and proteostatic dysfunction.

### Mechanical trauma induces a distinct transcriptional stress response

**Fig. 2:**
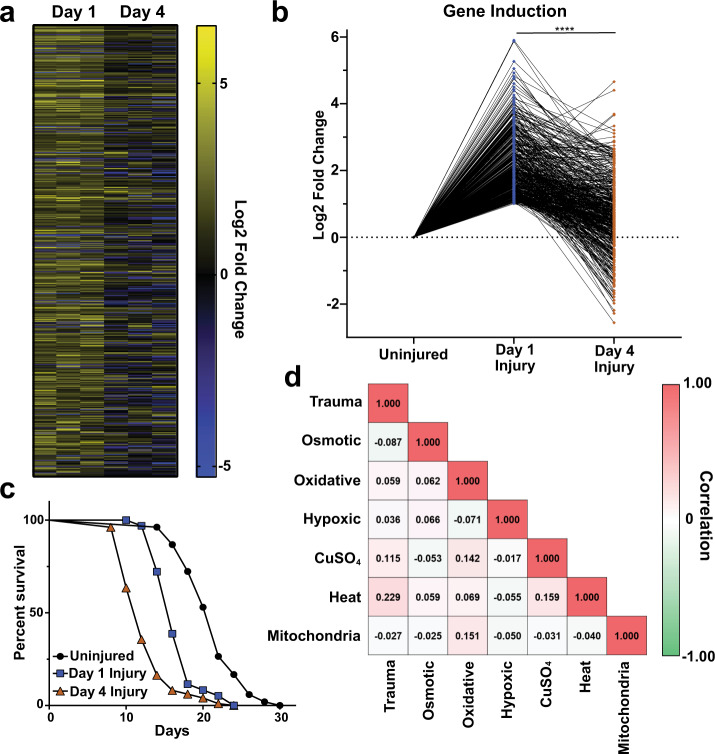
Mechanical stress activates a distinct age-dependent transcriptional response. **a** Genome-wide transcriptional analysis of worms subjected to trauma at Day 1 or 4 of adulthood. Log_2_ fold-change reported versus age-matched controls. Heat map displays 502 genes significantly regulated one hour after trauma in Day 1 adult worms (fold-change >2, *p* < 0.05, multiple two-sided *t*-tests), *n* = 3 biological replicates. Scale, yellow and blue intensity correspond to log_2_ fold change in transcriptional activation and repression, respectively. **b** Log_2_ fold-change trend of stress-activated transcripts at Day 1 (blue) and Day 4 (brown) of adulthood. Each gene was normalized to age-matched controls (Uninjured). ****p < 0.0001, two-sided *t*-test. **c** Worm lifespan comparing no injury (black circles) to moderate, paralytic injury at Day 1 (blue squares) or Day 4 (brown triangles) of adulthood. See Supplementary Table 1. **d** Cross-correlation matrix comparing induction of genes by mechanical stress to induction of those genes by other published stressors in *C. elegans*. The correlation coefficient for each condition is denoted in the corresponding box. Scale, red and green intensity correspond to positive and negative correlation, respectively.

To examine the cellular response occurring in *C. elegans* immediately after blunt force injury, we performed genome-wide transcriptional analysis via microarray on animals injured at day 1 or day 4 of adulthood. Compared to age-matched non-injured animals, we identified 437 genes upregulated and 113 genes downregulated by mechanical stress (Fig. 2a). Upregulated transcripts fell into several protein classes, including hydrolases (34 genes), ion-binding proteins (73 genes), oxidoreductases (17 genes), phosphotransferases (19 genes), transcription factors (19 genes), transmembrane signaling receptors (22 genes), and transmembrane transporters (23 genes) (Supplementary Data 1). As reported for other stress response pathways^39–41^, transcriptional activation of this mechanical stress response was blunted in older animals compared to age-matched, non-injured controls (Fig. 2a, b). Transcripts significantly activated or repressed by trauma clustered into six groups based on their age-dependent regulation. Of note, only 159 of 437 genes were upregulated >2-fold in both day 1 and day 4 animals (Supplementary Fig. 2a–c) and the majority of these genes were induced to a lesser extent in day 4 worms. Transcripts downregulated by stress followed similar trends (Supplementary Fig. 2d–f). Coinciding with blunted transcriptional activity with age, worms exhibited a shorter lifespan when injured later in adulthood compared to early adulthood (Fig. 2c, Supplementary Table 1).

To investigate this uncharacterized transcriptional response, we utilized gene ontology enrichment analysis. Significantly overrepresented gene-ontology (GO) terms included biological processes related to stress response, immune function, cell killing, and neuropeptide signaling (Supplementary Fig. 2g). The only significantly enriched molecular functions related to metal ion binding (Supplementary Fig. 2h). Enriched cellular components included membrane components, extracellular proteins, and the intraciliary transport particle B, which is important for the maintenance of *C. elegans* neuronal sensory cilia (Supplementary Fig. 2i)^42^. Furthermore, phenotype enrichment analysis identified several gene-related phenotypes (Supplementary Fig. 2j) primarily related to stress conditions. Taken together, enrichment analysis revealed that mechanical stress caused by blunt force injury activates a multifactorial set of genes including many known stress-responsive genes associated with oxidative stress, heavy metal stress, and immune activation.

Organisms are constantly challenged in an ever-changing environment. Highly specialized adaptive responses to these challenging conditions both maintain viability during that particular stress and facilitate recovery. Thus, organisms possess a multitude of transcriptional responses tailored to different stress conditions^43^. To determine if blunt force trauma activates a distinct stress response, we compared the transcriptional profile of injured worms to other known stress responses in *C. elegans*. Cross-correlation analysis revealed that no reported stress response in *C. elegans* alone accounts for the transcriptional activity elicited by mechanical stress (Fig. 2d). Of the analyzed stresses, heat shock and copper sulfate stress were the most similar, agreeing with GO term analysis that identified heavy metal response variants, ion-binding proteins, and innate immune responders, but were still poorly correlated with mechanical stress (*r* = 0.23 and 0.12 respectively). Thus, the transcriptional regulation occurring immediately after trauma represents a distinct and reproducible gene set that exhibits age-dependent activation.

### Screening of stress-responsive genes identifies several MAPK signaling factors as modulators of trauma-induced dopaminergic degeneration

**Fig. 3:**
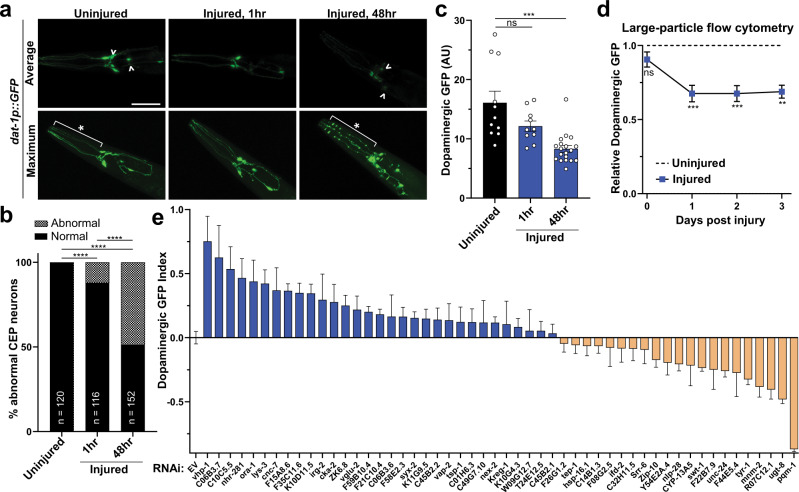
Reverse genetic screening of stress-responsive genes reveals several modulators of neurodegeneration. **a** Confocal fluorescence micrographs of transgenic worms (PMD13) expressing GFP in dopaminergic neurons (*dat-1p::GFP*). Images depict uninjured worms and 1 or 48 h post injury. Upper panels, average-intensity projections of z-stacks show relative brightness. ^ differences in neuronal GFP retention. Lower panels, maximum-intensity projections of Z-stacks show neuron morphology. *Neuronal beading after trauma. Reference Fig. 3b, c. Scale = 50 µm. **b** Quantification of CEP neuronal damage without injury and 1 or 48 h post injury. Neurons with visual beading, dendritic loss, or cell-body loss were scored as abnormal (checkered pattern). ****p < 0.0001 by chi-squared test. *n*-value represents number of worms examined. **c** Quantification of fluorescence intensity from average projections of CEP neuron z-stacks, uninjured (black) and 1 and 48 h post injury (blue). Mean + SEM, ns (not significant), and ***p = 0.0003 by one-way ANOVA with Dunnett test. *n* denotes animals from three independent trials, *n* = 11 (Uninjured), *n* = 10 (1 h injury), *n* = 19 (48 h injury). **d** Large-particle flow-cytometry of worms, expressing GFP in dopaminergic neurons, subject to no injury (---) or paralytic 8600 rpm injury (blue square). Fluorescence intensity for each time-point normalized to the uninjured fluorescence intensity of age-matched controls. Mean ± 95% CI. Left to right, ***p = 0.0008 and 0.0007, **p = 0.0030. Mixed-effects analysis with Bonferonni’s multiple comparisons test. See Supplementary Data 5 for additional statistics. **e** Large-particle flow cytometry screening results of dopaminergic neuronal health following RNAi treatment and two days post trauma. *X*-axis, RNAi gene target or empty vector control (EV). *Y*-axis, Dopaminergic GFP index indicates relative loss of *dat-1p::GFP* signal of injured worms from each RNAi condition compared to injured worms grown on EV bacteria. Mean ± 95% CI. Positive values (blue) indicate reduced loss of fluorescence when compared to age- and RNAi-matched uninjured controls. A value of one indicates no change of fluorescence compared to uninjured controls. Negative values (tan) represent increased loss of fluorescence. See Supplementary Data 5 for statistics.

When confronted with blunt force injury, the aging analysis indicated that inefficient activation of this mechanical stress response may have deleterious consequences on organismal health. Whether this set of stress-responsive genes affected cellular health following trauma was unclear. To this end, we established a targeted screening method to determine whether genetic knockdown of mechanically responsive transcripts could affect cell viability. Based on our neurological dysfunction analysis, we hypothesized that blunt force trauma induces neuronal degeneration and/or death in *C. elegans*. As an established model for neurodegeneration in *C. elegans*, transgenic expression of GFP exclusively within dopaminergic neurons, *dat-1p::GFP*, enables analysis of neuronal death through GFP fluorescence retention^44^. Compared to non-injured controls, dopaminergic neurons from injured worms displayed phenotypes characteristic of neurodegeneration and death at 48 h post-injury, as evidenced by dendritic GFP beading and/or the loss of GFP retention in processes and the soma (Fig. 3a, b). However, only 12% of worms displayed morphologic defects in dopaminergic neurons within one hour of injury, reaffirming that blunt force trauma differs from nerve transection models (Fig. 3a, b).

To adapt this model of trauma-induced dopaminergic neurodegeneration as a screening tool, we sought to increase throughput by analyzing the dopaminergic GFP signal by large-particle flow cytometry. This enables near-simultaneous detection and selection of thousands of living, intact animals. By confocal microscopy, we observed that worms displayed a loss of dopaminergic GFP intensity by 48 h post injury (Fig. 3a, c). Furthermore, loss of GFP signal was significantly correlated with the proportion of abnormal or damaged dopaminergic CEP neurons (*r* = 0.71, Supplementary Fig. 3a). Consistent with microscopy, time-course analysis of injured animals by large-particle flow cytometry showed a significant loss of GFP fluorescence beginning 24 h post injury, which persisted through the 72 h post injury period (Fig. 3d). Injured animals isolated from the bottom 10th percentile of dopaminergic GFP intensity by flow cytometry displayed predominately missing or grossly abnormal neurons (Supplementary Fig. 3b). Conversely, injured animals from the top 10th percentile of dopaminergic GFP intensity contained healthy dopaminergic neurons upon visual inspection by fluorescence microscopy (Supplementary Fig. 3b). To further validate flow cytometry analysis of dopaminergic neurodegeneration, worms treated with the dopaminergic neurotoxin MnCl_2_^45^ likewise exhibited reduced fluorescence signal (Supplementary Fig. 3c). Therefore, dopaminergic GFP signal intensity by large-particle flow cytometry correlated with the overall health of the dopaminergic neurons.

Utilizing these transgenic animals expressing GFP in dopaminergic neurons coupled with our model of blunt force trauma and automated large-particle flow cytometry, we performed a targeted genetic screen. With 480 differentially upregulated transcripts after trauma, potential gene targets were filtered using the following criteria at the time of the screen: (1) possession of human sequence similarity by BLAST or homology annotated on WormBase, (2) a minimal transcript abundance cutoff, and (3) expression in the nervous system. From this analysis, 89 genes fulfilled our established criteria, of which 51 possessed valid clones in the *C. elegans* RNAi library (Supplementary Data 2–4).

Following genetic knockdown by RNAi during development, worms received a moderate injury at day 1 of adulthood and were subsequently scored for dopaminergic neurodegeneration by flow cytometry 48 h post trauma. Neurodegeneration was scored as a loss of GFP signal (indicative of fluorescence retention in the neuron) compared to age- and RNAi-matched uninjured controls, and normalized to the loss of GFP signal observed in injured animals grown on control vector (EV) RNAi in each independent experiment. This value was termed the “dopaminergic GFP index”. From this screen, we identified significant neuroprotection by individual knockdown of 15 stress-activated genes. Conversely, knockdown of seven other genes significantly increased neurodegeneration (Fig. 3e, Supplementary Data 5).

Several of these genes have noted effects in other models of neurodegeneration and cell stress. Knockdown of the stress-inducible HSP70 molecular chaperone, F44E5.4, exacerbated neurodegeneration after blunt force injury, consistent with rodent studies where HSP70 levels directly correlated with neuroprotection after controlled cortical impact (CCI) brain injury^46^. Interestingly, trauma did not alter the expression of the well-characterized heat-activated small heat shock protein, HSP-16.2^47^, suggesting a distinct role for this particular HSP70 in mechanical stress. Moreover, UDP glucuronosyltransferase (*ugt-8*) has been noted to be transcriptionally activated in models of brain and liver injury^48,49^ and may act as a protective response against toxic metabolites. Inhibition of the C10C5.5 mouse homolog, aminoacylase, protected cortical neurons against neurotoxic compounds^50^. Lastly, expression of dopachrome tautomerase (*tyr-1*) was found to be cytoprotective against dopamine and hydroquinone toxicity^51^. Furthermore, unique homologs/analogs of *C. elegans* genes meeting our screening criteria were overrepresented (4.5-fold, *p* = 0.0002) in upregulated genes (8/23 annotated in both sets) from microarray analysis of mouse traumatic brain injury via CCI (Supplementary Fig. 3d, Supplementary Data 6).

Consistent with MAPK activation after head trauma in rodents^52,53^, MAPK-associated components represented the largest group of screening candidates, comprising 14% of the screened genes. These genes include *irg-2*, *cyp-13A5*, *zip-5*, *zip-10*, and *vhp-1*^54–57^ in addition to targets of the KGB-1 Jun kinase, *lys-3* and *kreg-1*^58^. Axotomy models have established the role of MAPK components and the MAPK phosphatase, *vhp-1*, in the regeneration of GABAergic neurons^28,29^.

### VHP-1 is a stress-inducible phosphatase that regulates mechanical stress-activated transcription and neurodegeneration

**Fig. 4:**
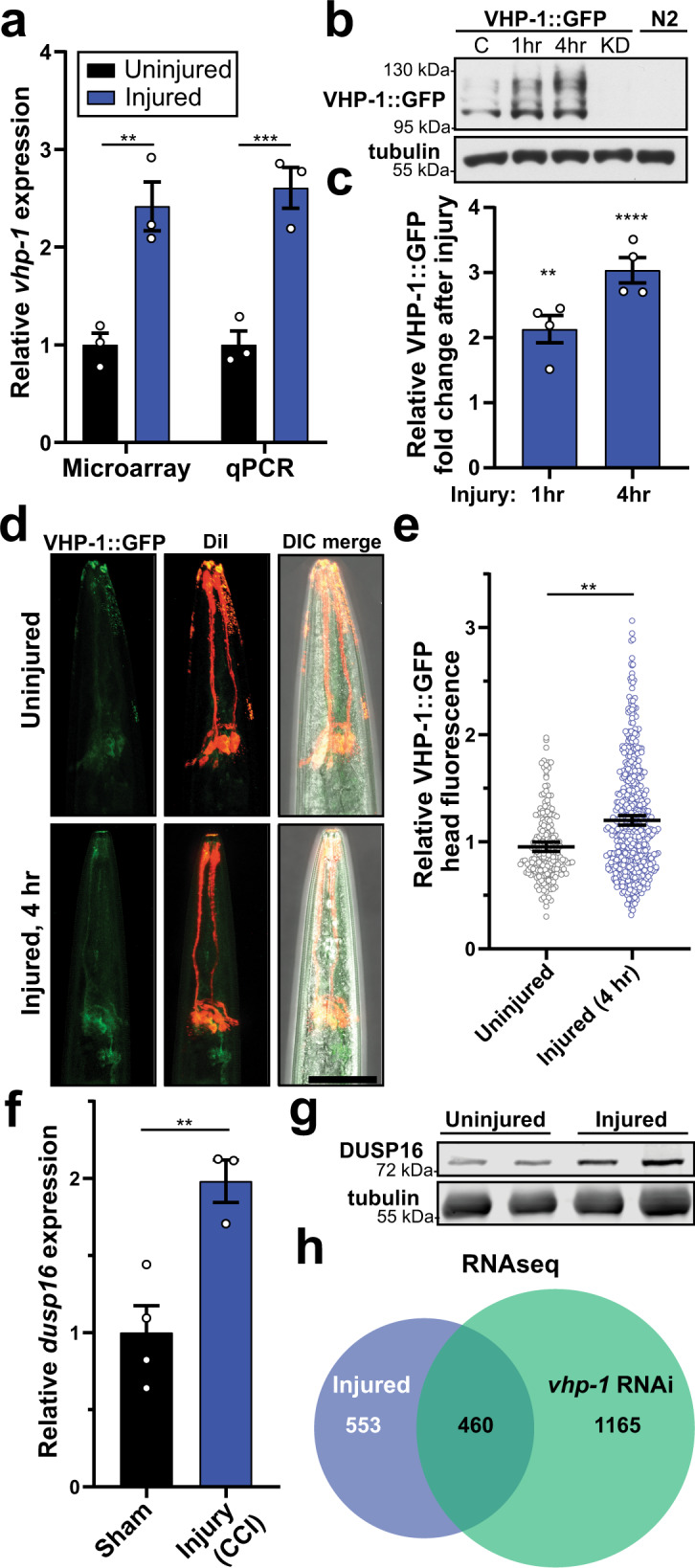
VHP-1 is a stress-inducible phosphatase in response to blunt force trauma. **a** Microarray and qPCR showing relative transcript abundance of *vhp-1* in uninjured (black) and injured (blue) conditions. Mean ± SEM, **p = 0.0014, ***p = 0.0006 by two-way ANOVA with Tukey test, *n* = 3, points denote mean biological replicates. **b** Western blot of worms expressing VHP-1::GFP either uninjured “C”, 1 or 4 h post-injury. KD, *vhp-1* RNAi knockdown. N2 worms lack transgene. (top) anti-GFP, (bottom) anti-tubulin. **c** Quantification of VHP-1::GFP by western blots, relative to VHP-1::GFP band intensity from uninjured worms. Mean ± SEM, **p = 0.0017 and ****p < 0.0001 by one-way ANOVA with Dunnett test, *n* = 4 biological replicates. **d** Confocal fluorescence micrographs of transgenic worms expressing VHP-1::GFP. Amphid sensory neurons are stained red with DiI for reference. Images depict uninjured worms (upper panels) and 4 h post injury (bottom panels). Reference Fig. 4e. Scale = 50 µm. **e** Quantification of VHP-1::GFP by large-particle flow cytometry comparing fluorescence in the worm head either uninjured or 4 hours (h) post injury. Mean + SEM, **p = 0.0022 by two-sided unpaired *t*-test. Uninjured (gray circles), *n* = 201 animals and Injured 4 h (blue circles), *n* = 503 animals over two independent trials. **f** Relative transcript abundance by microarray of mouse *Dusp16* 4 hours (h) following controlled cortical impact (CCI) compared to sham surgery and 4 h post-surgery. Mean ± SEM. **p = 0.0089 by two-sided unpaired *t*-test. *n* = 4 (sham), 3 (CCI). From GDS2850. **g** Anti-DUSP16 western blot from uninjured mice or mice receiving closed-head, concussive injury by linear impact via rail-guided weight drop. Tubulin, loading control. **h** Venn-diagram from paired-end RNAseq comparing the transcriptional response of EV-injured/EV-uninjured worms (blue) versus *vhp-1* RNAi/EV (green), fold-change ≥2, FDR *p* ≤ 0.05, Baggerly’s test. EV *n* = 3, *vhp-1* RNAi *n* = 2.

RNAi targeting of the dual-specificity MAPK phosphatase, *vhp-*1, proved the most neuroprotective in our screen (Fig. 3e). Transcript analysis through qPCR confirmed a 2.6-fold increase in *vhp-1* transcript abundance one hour after injury (Fig. 4a). Western blot analysis of a VHP-1*::*GFP transgene^59^ confirmed that VHP-1 protein levels were elevated within an hour of trauma and continued to rise at four hours (Fig. 4b, c). Within the head of the animal, levels of VHP-1::GFP are elevated 4 h after trauma (Fig. 4d, e). Likewise, multiple models of mammalian head trauma implicate regulation of the mammalian VHP-1 homolog, DUSP16. From previous mouse brain injury datasets^60^, we found that *Dusp16* was transcriptionally upregulated within four hours post injury (Fig. 4f). Furthermore, DUSP16 protein was elevated in total brain tissue two hours post trauma in our mouse model of closed-head concussive injury (Fig. 4g), suggesting shared transcriptional and translational control in mice or worms subjected to blunt trauma. Interestingly, RNAseq analysis from worms given trauma or treated with *vhp-1* RNAi revealed that nearly half (460/1013) of the genes significantly upregulated by trauma (>2 fold, FDR *p* ≤ 0.05) were also upregulated upon *vhp-1* knockdown (Fig. 4h), suggesting an important regulatory role for VHP-1 in the transcriptional response to mechanical stress or preconditioning of animals to better sustain trauma. Enrichment analysis of these 460 shared transcripts revealed genes involved in cadmium stress-response, innate immunity, metal ion binding, neuronal signaling, and locomotion (Supplementary Fig. 4a–c).

**Fig. 5:**
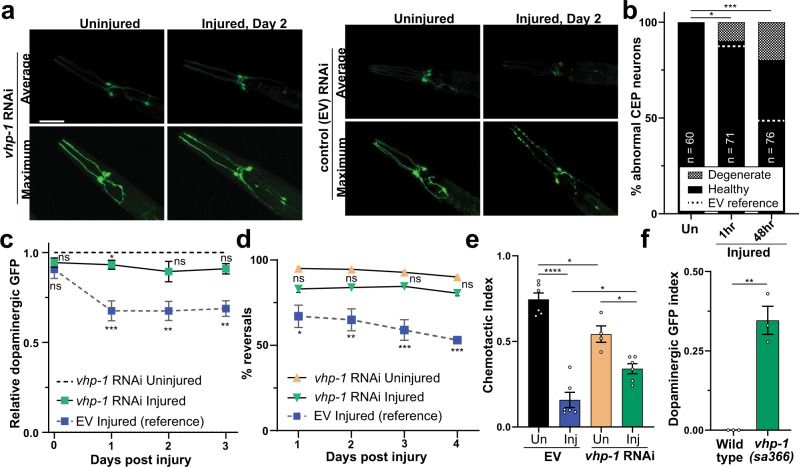
Reducing VHP-1 expression suppresses trauma-induced neurodegeneration. **a** Confocal micrographs of GFP expressing dopaminergic neurons with and without injury and *vhp-1* RNAi. Shown are average or maximum projections of Z-stacks. Reference Fig. 5b, c. Scale = 50 µm. **b** Quantification of CEP neuronal phenotype from *vhp-1* RNAi maximum projections. *vhp-1* RNAi uninjured (Un) versus injured *p = 0.0124, ***p = 0.0003. *vhp-1* RNAi versus empty vector (EV, dashed lines) 1 and 48 hour (h) post injury, ns (not significant) *p* = 0.8122, and ****p < 0.0001 respectively, chi-squared test. **c** Large-particle flow cytometry time-course comparing effects of dopaminergic GFP retention in injured *vhp-1* RNAi (green squares) to uninjured *vhp-1* RNAi (---). Injured empty vector (EV, blue squares) reference from Fig. 3D, gray dashed lines. Mean ± 95% CI. Mixed-effects analysis with Tukey’s multiple comparisons test. See Supplementary Data 5 for statistics. **d** Nose-touch response of uninjured (tan upright triangles) or injured (green inverted triangles) worms on *vhp-1* RNAi. Injured empty vector (EV, blue squares) reference from Fig. 1J (gray dashed lines) Mean ± SEM. See Supplementary Data 5 for statistics. **e** Chemotaxis of uninjured (Un, black and tan) or injured (Inj, blue and green) worms on empty vector (EV, black and blue) or *vhp-1* RNAi (tan and green) with butanol 48 h post injury. Chemotactic index of 1.0 indicates 100% preference for butanol and 0.0 indicates no preference. Mean ± SEM, biological replicates denoted. See Supplementary Data 5 for statistics. **f** Large-particle flow cytometry results of wild type, PMD13, and *vhp-1(sa366)* mutants, PMD153, in green two days post trauma. Dopaminergic GFP index indicates relative loss of *dat-1p::GFP* signal in injured *vhp-1(sa366)* mutants (green) compared to injured wild type. Mean ± 95% CI. **p < 0.0015, two-sided paired *t*-test, *n* = 1902 for wild type and *n* = 1342 for *vhp-1(sa366)* over three independent trials. Positive values indicate reduced loss of fluorescence when compared to age-matched uninjured controls. A value of one indicates no change of fluorescence compared to uninjured controls.

Fluorescence confocal microscopy and flow cytometry time-course analysis verified the protective effects of *vhp-1* RNAi on dopaminergic neurons (Fig. 5a–c). Compared to the EV control, *vhp-1* knockdown largely rescued the dopaminergic degenerative phenotype observed 48 hours after trauma (Fig. 5a–c), and suppressed paralysis immediately following trauma (Supplementary Fig. 5a). Moreover, trauma-induced deficits in mechano-sensation, chemo-sensation, and motility were suppressed by *vhp-1* RNAi (Fig. 5d, e, Supplementary Fig. 5b). Consistent with the effects of *vhp-1* RNAi, neuroprotection was also observed after trauma in the *vhp-1(sa366)* hypomorphic mutant strain (Fig. 5f).

Regarding its role in the nervous system, *vhp-1* knockdown in a neuronal-specific RNAi worm strain, which ectopically expresses SID-1 exclusively in neurons of an otherwise *sid-1* null animal^61^, rescued degeneration of dopaminergic neurons (Supplementary Fig. 5c). However, muscle-specific knockdown of *vhp-1* had no effect on GFP retention in dopaminergic neurons after trauma, further suggesting a role for neuronal VHP-1 (Supplementary Fig. 5d). Ectopic VHP-1::mCherry expression levels in the nervous system inversely correlated with dopaminergic GFP retention after trauma (Supplementary Fig. 5e). Moreover, we generated an alternate RNAi targeting the 3’ untranslated region (3’UTR) of *vhp-1* transcripts, which enabled selective knockdown of the endogenous *vhp-1* but not transgenic VHP-1::mCherry in neurons due to its *unc-54* 3′UTR. While this *vhp-1* 3’UTR RNAi was neuroprotective in the absence of neuronal VHP-1::mCherry, it ceased to suppress dopaminergic degeneration in the presence of the RNAi-resistant, neuronal VHP-1::mCherry (Supplementary Fig. 5f–h). Thus, VHP-1 is a trauma-inducible phosphatase that negatively regulates transcriptional programs related to mechanical stress and whose knockdown in the nervous system is sufficient for neuroprotection.

### A negative feedback loop via KGB-1 and the AP-1 complex regulates VHP-1 stress induction

**Fig. 6:**
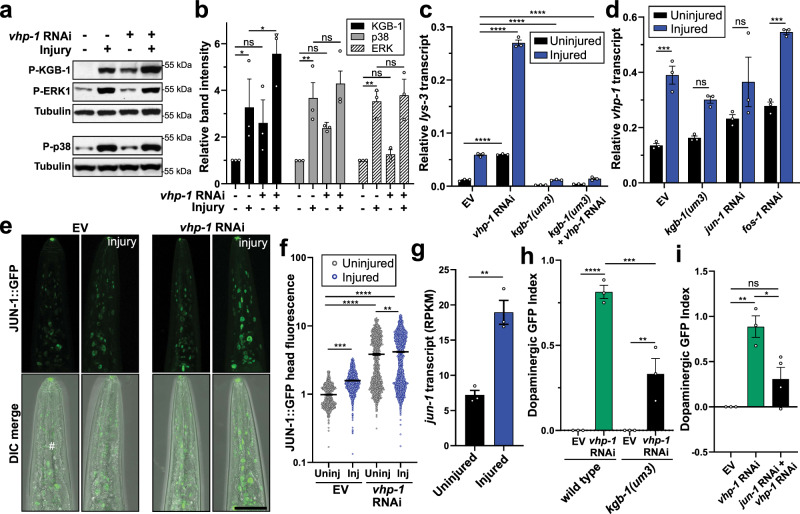
Bidirectional regulation between VHP-1, KGB-1, and JNK signaling influences neurodegeneration following mechanical stress. **a** Western blot of phosphorylated (P-) KGB-1, p38, and ERK in response to injury and/or *vhp-1* RNAi. Tubulin, loading control. **b** Quantification of phospho-specific western blots relative to uninjured empty vector. Mean + SEM. From left to right, ns (not significant), *p = 0.0116, *p = 0.0109, **p = 0.0031, **p = 0.0049 by two-way ANOVA with Tukey test. *n* = 3 biological replicates. KGB-1 (black), p38 (gray), ERK1/2 (stripes). **c**, **d** qPCR of *lys-3* or *vhp-1* transcript abundance either uninjured (black) or 1 h post injury (blue) in wild type or *kgb-1(um3)* mutants with the respective RNAi. Mean ± SEM. ****p < 0.0001, ***p = 0.0004 (EV) and ***p = 0.0002 (*fos-1* RNAi) by two-way ANOVA with Tukey test. *n* = 3 biological replicates. **e** Confocal micrographs of JUN-1::GFP expression in the worm head with and without *vhp-1* RNAi and injury (4 h post trauma). Differential interference contrast, DIC. Reference Fig. 6f. Scale = 50 µm. **f** Quantification of JUN-1::GFP by large-particle flow cytometry comparing fluorescence in the worm head for uninjured (Uninj, gray) and 4 h post injury (Inj, blue) with and without *vhp-1* RNAi. Mean ± SEM. Two-way ANOVA with Tukey multiple comparison test. See Supplementary Data 5 for statistics. **g** JUN-1 transcript abundance determined by RNAseq without injury (black) and 1 h post injury (blue). Mean ±  SEM. **p = 0.003 by two-sided unpaired *t*-test. *n* = 3 biological replicates. Reads per kilobase million (RPKM). **h**, **i** Large-particle flow cytometry showing the effects of trauma on dopaminergic GFP retention 48 h post injury. Shown are values indexed to empty vector control (EV). Mean ± SEM. **h** *vhp-1* RNAi in wild type (green) or *kgb-1(um3)* mutants (black). **i** effects of *jun-1* RNAi with *vhp-1* RNAi (black) compared to *vhp-1* RNAi alone (green). Positive values indicate reduced loss of fluorescence when compared to age-matched uninjured controls. A value of one indicates no change of fluorescence compared to uninjured controls. See Supplementary Data 5 for statistics.

VHP-1 and human homologs DUSP8/16 have previously been implicated as JNK- and p38-specific MAP Kinase phosphatases^59,62^. We sought to understand its role in the context of mechanical stress and trauma-induced neurodegeneration. Consistent with mammalian models of head trauma^52,53,63^, the three canonical MAPK pathways, ERK (MPK-1), p38 (PMK-1), and JNK (KGB-1) were activated in *C. elegans* as evidenced by increased phosphorylation within minutes following trauma (Fig. 6a, Supplementary Fig. 6a). Consistent with cross-correlation analysis implicating similarities between mechanical stress and copper sulfate treatments (Fig. 2d), KGB-1 activation via phosphorylation in worms has been reported under copper stress conditions^64^. Upon *vhp-1* RNAi, both KGB-1 and PMK-1 showed increased basal phosphorylation. However, *vhp-1* knockdown induced KGB-1 phosphorylation to the greatest extent and had no effect on MPK-1, agreeing with findings that VHP-1 preferentially regulates specific MAPKs^29,59,65^. Furthermore, only KGB-1 displayed significantly increased phosphorylation following mechanical trauma with *vhp-1* knockdown (Fig. 6a, b). In agreement, recombinant VHP-1 can directly hydrolyze phosphate from KGB-1^59^.

Out of the top 250 genes regulated in *kgb-1(km21)* mutant worms^66^, 69 were significantly regulated by *vhp-1* RNAi (FDR *p* ≤ 0.05) as determined by RNAseq. Regulation of these genes by either KGB-1 or VHP-1 showed a significant negative correlation (*r* = −0.58, *p* < 0.0001, Supplementary Fig. 6b), indicating that these factors operate in opposition. In support, a known KGB-1 transcriptional target, *lys-*3, was upregulated by mechanical stress (Supplementary Data 3). To further validate KGB-1 activation, we confirmed with qPCR analysis that *lys-3* was transcriptionally activated and that its expression required KGB-1 (Fig. 6c). Moreover, *vhp-1* RNAi was sufficient to increase *lys-3* activation in a KGB-1-dependent manner, further confirming its repressive role for KGB-1 (Fig. 6c).

Previous reports suggest that KGB-1 may be involved with the developmental expression of a *vhp-1* isoform, *vhp-1a*, as evidenced by increased *vhp-1a* upon *vhp-1* RNAi^66^. We were interested if acute *vhp-1* transcript upregulation by trauma represented a stress-induced negative feedback loop on KGB-1 activity. Interestingly, transcript abundance of *vhp-1* in adulthood was not significantly changed in *kgb-1(um3)* mutant animals (Fig. 6d), indicating that basal *vhp-1* transcript levels are independent of KGB-1 function in adults. However, significant induction of *vhp-1* transcription upon injury required KGB-1 and the AP-1 transcriptional complex subunit, JUN-1 (Fig. 6d, Supplementary Fig. 6c). At the protein level, *kgb-1* RNAi modestly reduced basal VHP-1::GFP levels in the animal head (Supplementary Fig. 6d, e), suggesting a role for translational or post-translational regulation of VHP-1. In addition to *vhp-1* induction, steady-state protein levels of JUN-1::GFP were elevated upon injury and *vhp-1* RNAi (Fig. 6e, f). Moreover, *jun-1* transcription was elevated 1-hour post injury (Fig. 6g). Altogether, these data indicate that VHP-1 acts to abrogate stress-induction of JNK signaling in the context of mechanical injury, and that stress-induced phosphorylation of KGB-1 acutely activates *vhp-1* transcription through AP-1 in a negative feedback process.

### VHP-1-mediated protection requires MAP3K signaling through DLK-1, MLK-1, and downstream KGB-1 and JUN-1

We hypothesized that the neuroprotection due to *vhp-1* knockdown may be the result of increased activity of *vhp-1* targets. To determine whether these protective effects required *kgb-1* or *jun-1*, we co-administered RNAi targeting both *vhp-1* and either gene in tandem. Knockdown of any single or dual combination of JNK or p38 gene with *vhp-1* failed to significantly abolish the neuroprotective phenotype (Supplementary Table 2). However, deducing genetic pathways based on partial reduction of gene function can be misleading due to increased false discovery caused by potential off-target effects of the RNAi. Thus, we examined trauma-induced neurodegeneration in a *kgb-1(um3)* mutant and observed that neuroprotection by *vhp-1* RNAi was dependent upon *kgb-1* (Fig. 6h). Moreover, *vhp-1-*mediated neuroprotection also required *jun-1* (Fig. 6i, Supplementary Fig. 6f), indicating that these protective effects involve transcriptional regulation of factors downstream of MAPK signaling. *C. elegans* contain five potential JNKs and three p38s. Previous studies have suggested that significant interaction occurs between the branches of MAPK signaling^67^ which, in turn, complicates epistasis experiments at the MAPK level. However, *C. elegans* represent a simplified system to study upstream MAPK components, which directly phosphorylate and activate downstream MAPKs, when compared to mammals^68^.

We examined the effects of RNAi targeting MAP2Ks in the worm, including *mek-1, mkk-4, and jkk-1*. MEK-1 and MKK-4 in particular have been reported to be required for axonal regeneration following axotomy;^33^ however, knockdown of any single MAP2K with *vhp-1* failed to abrogate neuroprotective effects (Supplementary Table 2). Similarly, we examined the effects of RNAi targeting known stress-activated MAP3Ks including *mlk-1, dlk-1*, and *nsy-1*. In axotomy models of neuronal regeneration, *dlk-1* knockout or knockdown prevented axon regeneration^33,69^. Yet, *dlk-1* RNAi administered alone had no effect on neuroprotection against trauma by *vhp-1* RNAi. Rather, tandem RNAi knockdown of *mlk-1* and *dlk-1*, but no other combination, abrogated the neuroprotective effects of *vhp-1* RNAi after trauma (Supplementary Table 2), indicating that these overlapping signaling cascades may confer some specificity to downstream substrates. Consistent with the requirement for both *mlk-1* and *dlk-1*, transcriptional activation of *vhp-1* by trauma required both *mlk-1* and *dlk-1* but not *nsy-1* RNAi (Supplementary Fig. 6g). Likewise, *mlk-1* and *dlk-1* knockdown reduced, but did not abolish, transcriptional induction of *lys-3* (Supplementary Fig. 6h), indicating partial suppression of KGB-1 activity. Since *kgb-1*, its downstream target, *jun-1*, and its upstream MAP3K regulators *mlk-1* and *dlk-1* were required for both the neuroprotective effects of *vhp-1* RNAi as well as transcriptional induction of *vhp-1*, these data suggest a pathway from the level of MAP3K to MAPK to the AP-1 transcriptional complex.

### MATH-33, a stress-inactivated deubiquitinase, regulates VHP-1 protein abundance

Following activation, attenuation of stress-response machinery presents a critical step to reestablish cellular homeostasis and avoid deleterious consequences from chronic activity^70^. Such is the case for VHP-1, which we implicate as an inducible negative regulator of stress signaling; however, VHP-1 enhances trauma-induced cell death when overexpressed in the nervous system (Supplementary Fig. 5e). Moreover, ectopic overexpression of VHP-1 revealed its apparent lack of protein stability. Compared to similar transgenic constructs (PMK-1::mCherry) under the same 5′ promoter and 3′ UTR, transgenic expression of VHP-1::mCherry in all tissues or specifically in neurons appeared greatly reduced (Supplementary Fig. 7a). Combined with the observed emergence of slower migrating, immunoreactive VHP-1 bands by western blot (Fig. 4b), we hypothesized that VHP-1 may be processed and degraded through the ubiquitin-proteasome pathway as a means of attenuation. Attempts to enrich animals with elevated VHP-1::mCherry fluorescence in all tissues resulted in developmental arrest and/or premature death, further suggesting that chronic elevation of VHP-1 is deleterious to animal physiology.

**Fig. 7:**
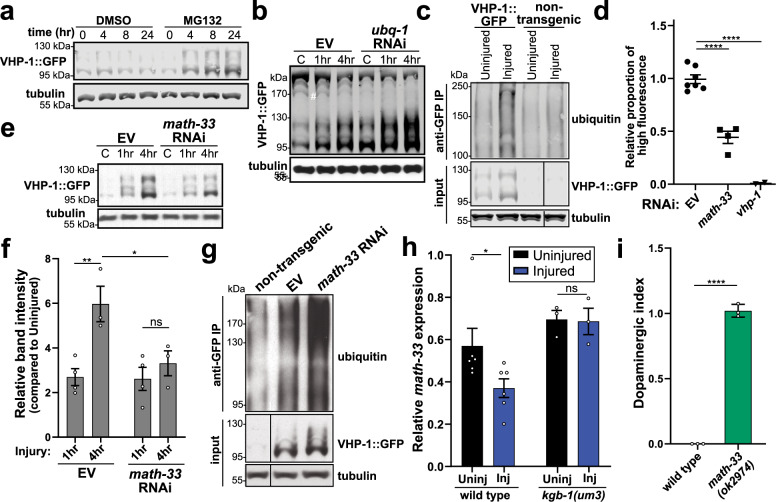
Post-translational regulation of VHP-1 stability by components of the ubiquitin-proteasome system. **a** Western blot time-course of VHP-1::GFP following treatment with DMSO (vehicle) or 50 µM MG132. Tubulin, load control. **b** Western blot of VHP-1::GFP without injury (control, C) or 1 and 4 hours (h) post injury. Worms treated with *ubq-1* RNAi for 24 h. ^#^*ubq-1* RNAi sensitive high-MW bands. **c** Co-immunoprecipitation of VHP-1::GFP immunoblotted with anti-ubiquitin. Uninjured and injured non-transgenic worms (N2) as control. Input, VHP-1::GFP and tubulin from total worm extracts. **d** Effects of *math-33* (deubiquitinase, squares) or *vhp-1* (triangles) RNAi on transgenic worms expressing VHP-1::mCherry in all tissues (PMD117). Flow cytometry data depicts the proportion of worms falling into a high-fluorescence gate. Data are proportional to EV control from each independent experiment. Mean ± SEM, ****p < 0.0001 by one-way ANOVA with Sidak test. **e** Western blot of VHP-1::GFP from worms before (C, control) or 1 and 4 hours (h) after injury treated with *math-33* RNAi. Tubulin, loading control. *n* = 4, uninjured and 1 h post injury, *n* = 3, 4 h post injury. **f** Quantification of western blots from Fig. 7e. Mean ± SEM. **p = 0.0088, *p = 0.0427, not significant (ns) *p* = 0.8134 by two-way ANOVA with Tukey test. **g** Co-immunoprecipitation of VHP-1::GFP (anti-GFP IP) from injured worms on empty vector (EV) or *math-33* RNAi immunoblotted with anti-ubiquitin. Injured, non-transgenic N2 worms as control. Input, VHP-1::GFP, and tubulin from total worm extracts. *n* = 4 biological replicates. **h** qPCR showing relative *math-33* transcript abundance in wild type and *kgb-1(um3)* mutant worms with (Inj) and without (Uninj) injury. Mean ± SEM, *p = 0.0135 by two-way ANOVA with Sidak test, WT *n* = 6, *kgb-1(um3)* *n* = 3. **i** Large-particle flow cytometry of wild type, PMD13, and *math-33(ok2974)* mutants, PMD173, two days post trauma. Dopaminergic GFP index indicates relative loss of *dat-1p::GFP* signal of injured *math-33(ok2974)* mutants compared to injured wild type worms. Mean ± 95% CI. ****p < 0.0001 by two-sided paired *t*-test. *n* = 4149, wild type, and *n* = 2601, *math-33(ok2974)*, over three independent trials. Positive values indicate reduced loss of fluorescence when compared to age-matched uninjured controls. A value of one indicates no change of fluorescence compared to uninjured controls.

Transgenic worms treated with the proteasome inhibitor, MG132, displayed VHP-1::GFP accumulation within 4 h of drug treatment, and the protein continued to accumulate throughout the 24 h time-course when compared to vehicle-treated worms (Fig. 7a). Alternatively, disruption of the ubiquitin-proteasome system through RNAi targeting ubiquitin, *ubq-1*, for 24 h increased steady-state levels of VHP-1::GFP both in uninjured worms as well as 1 to 4 hours after trauma (Fig. 7b). A reduction of higher molecular-weight, GFP-immunoreactive bands further accompanied *ubq-1* knockdown. Immunoprecipitation of VHP-1::GFP confirmed a substantial increase in ubiquitin material within 1 h of injury compared to both non-injured transgenic animals and non-transgenic controls (Fig. 7c). Furthermore, several distinct ubiquitin immunoreactive bands observed upon injury were of similar molecular weight to immunoreactive bands on anti-GFP blots.

With no previous reported links between VHP-1 or its mammalian homologs and particular ubiquitin machinery, we leveraged motif prediction algorithms to identify several potential ubiquitin ligase and hydrolase (deubiquitinase) binding motifs, which clustered within the C-terminus of the protein (Supplementary Fig. 7b). Based on these predictions, we knocked down the expression of candidate genes that were potentially homologous to or interacted with homologs of predicted mammalian binding partners. From our prediction analysis, we identified 13 ubiquitin ligases (*cul-1, sel-10, bath-43, mel-26, lin-23, cks-1, fzy-1, skpt-1, fsn-1, skr-5*, F10D7.5*, mfb-1*, and ZK1240.2) and the deubiquitinase (*math-33*) (Supplementary Fig. 7c). Utilizing the all tissue VHP-1::mCherry OE strain, we determined whether knockdown of the different E3 ubiquitin ligases or the ubiquitin hydrolase modulated steady-state protein levels of VHP-1, as measured by automated large-particle flow cytometry. We identified that knockdown of *math-33*, the homolog of mammalian USP7, decreased VHP-1 levels (Fig. 7d, Supplementary Fig. 7c).

RNAseq analysis identified *math-33* as a significantly downregulated gene one hour after trauma (Supplementary Fig. 7d). Western blot analysis of VHP-1::GFP revealed that *math-33* knockdown significantly reduced protein induction 4 h following trauma (Fig. 7e,f). Moreover, immunoprecipitation of VHP-1::GFP 1 h after injury revealed a near twofold increase in ubiquitinated VHP-1::GFP upon *math-33* RNAi (Fig. 7g, Supplementary Fig. 7e). Thus, suppression of *math-33* transcription upon mechanical stress may represent a means for accelerating VHP-1 protein turnover following accumulation. Having shown that trauma-induced activation of *vhp-1* required KGB-1 (Fig. 6d, Supplementary Fig. 6c), we sought to determine whether trauma-induced repression of *math-33* was also KGB-1 dependent. In support of RNAseq datasets, qPCR analysis revealed that trauma reduced *math-33* transcript levels (Fig. 7h). Furthermore, trauma-induced repression of *math-33* was abolished in the *kgb-1(um3)* strain, indicating that stress-induced repression of *math-33* depends on KGB-1 activity (Fig. 7h). Consistent with *math-33* RNAi reducing VHP-1::GFP levels 4 h post injury (Fig. 7e,f), trauma-induced loss of dopaminergic GFP signal was substantially reduced in *math-33(ok2974)* mutant animals (Fig. 7i). Therefore, with regards to the mechanical stress response, the ubiquitin-proteasome system modulates VHP-1 protein stability, and both stress-induced VHP-1 accumulation and neurodegeneration requires *math-33*, a deubiquitinase inhibited by KGB-1 following stress.

## Discussion

**Fig. 8:**
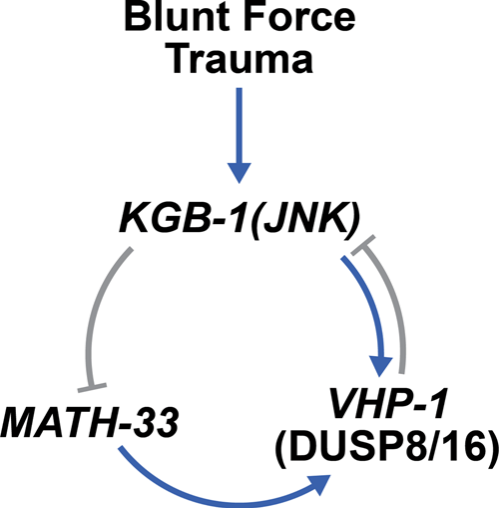
Dual feedback governs transcriptional and post-translational regulation of VHP-1. Mechanical stress via blunt force trauma activates KGB-1, which leads to the transcriptional upregulation of *vhp-1* and repression of *math-33*. VHP-1 is stabilized by MATH-33 and, in turn, represses KGB-1 activity.

We introduce a high-throughput model of blunt force trauma in the nematode, *C. elegans*, (Fig. 1a) and uncover distinct transcriptional activation and post-translational regulatory events. Through our calibrated stress paradigm, we characterize a dose-dependent paralytic response and identify an uncharacterized transcriptional program that correlates with age and lifespan determination. Leveraging this transcriptional framework, we perform a candidate-based reverse genetic screen and discover several stress-activated factors that modulate trauma-induced neurodegeneration. From this screen, we detail the molecular role and regulation of a stress-activated MAPK phosphatase, VHP-1, in relation to suppressing trauma-induced degeneration. We explore a complex genetic interaction between VHP-1 and several tiers of MAPK signaling, emphasizing the inherent complexity of kinase pathway interactions and the cellular response to blunt trauma. Further regulation by the ubiquitin-proteasome system and the *math-33* deubiquitinase culminates as multiple layers of negative feedback modulate VHP-1 activity (Fig. 8).

Utilizing the simple architecture of the nematode *C. elegans*, we introduce a model of blunt force injury by rapid multidirectional acceleration. Although randomized, thousands of animals are injured simultaneously, supporting reproducibility through probability. This is evidenced by a dose-dependent paralytic phenotype, highly reproducible chemo- and mechano-sensation defects, and consistent observation of dopaminergic neurodegeneration. Through large n-values and increased throughput via automated large-particle flow cytometry, we can investigate physiological and molecular details with greater statistical power. Gross examination of the musculature (Supplementary Fig. 1e–h) revealed limited damage and may prove to be an interesting tissue for future studies within our model. We chose to focus on the nervous system due in part to its complex cellular architecture, overrepresentation of cell number in *C. elegans* (302/959 cells), and established literature aiding the interpretability of results. The characterization of blunt trauma on other organ systems remains an outstanding question. Furthermore, while several sensory assays suggest dysfunction of other neuronal systems, the extent of degeneration of non-dopaminergic neuronal subtypes has yet to be determined.

Environmental fluctuations have favored the evolution of several stress response pathways, each tailored to manage a particular stress condition. Herein, we uncover a distinct transcriptional response to mechanical stress by blunt force trauma. Worms are a mobile soil-dwelling organism with the potential to experience several mechanical obstacles. Whether these environmental cues have favored the evolution of a mechanical stress response remains unclear. Notably, hermaphrodite worms possess 30 mechanosensory neurons compared to 2 thermosensory, 4 oxygen-sensing, and 2 osmosensory neurons^71,72^. These other neurons are associated with sensation of noxious stimuli and particular stress responses. We show some shared transcriptional features between mechanical stress and copper stress or heat shock by cross-correlation analysis (Fig. 2d). However, correlations between trauma and these stresses are low, indicating a distinct role for mechanical stress in gene transcription. Additional transcriptional analysis by RNAseq reveals that VHP-1 regulates 45% of all genes upregulated by our trauma model. Thus, the present work indicates a central role for VHP-1 and its targets in regulating the cellular response to mechanical stress. Interestingly, we find that genes expressed in mechanosensory neurons are overrepresented in the gene set activated by both trauma and *vhp-1* RNAi (Supplementary Fig. 4), indicating that VHP-1 likely possesses an important role in these neuronal subsets. Many significantly regulated genes lack annotation, highlighting the need to identify functions for these stress-responsive elements. Collectively, these findings establish a foundation on which to build a mechanistic understanding of the cellular response to blunt force trauma.

We discover that mechanical stress induces *vhp-1* transcription, yet its knockdown by RNAi paradoxically protects against trauma-induced neurodegeneration. This finding favors the concept that increased MAPK activity benefits cells following mechanical stress, similar to observations from other stress paradigms^73^. Indeed, every major branch of the MAPK signaling cascade is activated nearly instantaneously upon injury. Consistent with previous findings^59^, we find VHP-1 to primarily modulate KGB-1 activity following trauma, suggesting an important role for JNK signaling in the transcriptional response and neuroprotection. Consistent with cross-correlation analysis of mechanical stress with other defined stress paradigms, both copper and heat stress are associated with KGB-1 regulation^64,74^. KGB-1 has likewise been linked to axon regeneration^29^, supporting its role in the nervous system. Several studies have interrogated the roles of MAPKs in these regeneration paradigms. While our model of trauma-induced neurodegeneration also implicates MAPK signaling, we propose that there are distinct mechanistic differences between the two processes, agreeing with the concept of MAPK context specificity^75^. Of note, most regeneration studies focus on GABAergic neurons in *C. elegans*. It remains to be determined if different neuronal subtypes contain alternate mechanisms for protection or repair.

The MAPK cascade represents an interconnected network containing functional redundancy, which complicates the linkage of a single enzyme with a particular physiological effect. Emphasizing this point, *vhp-1* knockdown significantly upregulates transcription of multiple phosphatases, which likely contribute to the overall regulation of MAPK signaling. We find significant epistasis related to neuroprotection between VHP-1 and a single MAP kinase, KGB-1. Moreover, VHP-1, MLK-1, and DLK-1 comprise a three-way epistatic interaction. The combination of signaling through these MAP3Ks likely converges on the Jun kinase, KGB-1, and accounts for the neuroprotective effects of *vhp-1* knockdown and strongly suggests a requirement for MAPK activity to promote cell survival following trauma. Previous reports have likewise highlighted stress-resistance upon developmental *vhp-1* RNAi^65^. Interestingly, while stress-resistance in these paradigms requires the FOXO-transcription factor (*daf-16*), our studies do not support this requirement in the context of blunt trauma. Rather, enhanced activity downstream of KGB-1 and its AP-1 transcriptional complex subunit, *jun-1*, likely accounts for our observed neuroprotective phenotype (Fig. 6h,i). Everything considered we hypothesize that *vhp-1* RNAi preconditions the nervous system to sustain these traumatic events through basal activation of JNK signaling (Fig. 6). Thus, preemptive activation of this neuroprotective stress response may immediately mitigate cellular damage and promote survival. While we have not implicated transcriptional targets contributing to neuroprotection, these findings would be of interest to future studies detailing genetic regulation and molecular function of stress-activated genes.

Supporting our findings of ubiquitin-mediated regulation of VHP-1, E3 ligases and deubiquitinases are implicated as regulatory elements of the stress response and MAPK signaling^76–80^. While post-translational regulation of deubiquitinases has been extensively characterized^81^, few studies have examined their regulation at the transcriptional level^82–84^, and no studies have described deubiquitinases as transcriptionally regulated stress response elements themselves. The USP7 *C. elegans* homolog, MATH-33, is an important regulator of the FOXO transcription factor, DAF-16^85^. We demonstrate that mechanical stress represses transcription of *math-33*, and that *math-33* is necessary for stabilization of VHP-1 protein upon stress. In particular, trauma represses *math-33* in a KGB-1-dependent manner. Importantly, the removal of math-33 protects against trauma-induced neurodegeneration (Fig. 7i). Publicly available ChIP-seq datasets reveal JUN-1 and DAF-16 binding peaks upstream of *math-33* (WormBase, release WS274), indicating direct transcriptional control by KGB-1-associated transcription factors. Dysregulation of deubiquitinases has been linked to several human diseases including cancers and neuropathologies^81,86,87^. Due to its association with KGB-1 and VHP-1, we implicate an additional role for MATH-33. Namely, this genetic circuit acts as an additional layer of negative feedback to modulate MAPK suppression in response to mechanical trauma. Lastly, we propose that this auto-regulatory mechanism applies to a broad range of other MAPK paradigms. To date, the inherent complexity of such systems has hindered experimental interrogation.

## Methods

### *C. elegans* strains

All strains were maintained at 15 °C on an OP50 lawn grown on standard NGM plates. Age-synchronization was performed by hypochlorite treatment of gravid animals to obtain eggs. AGD1651 worms *(unc-119(ed3) III; uthSi7[myo3p::LifeAct::mRuby::unc-54 3’UTR* + *unc-119(+)] IV)* were received from Dr. Andrew Dillin. KM20 worms *(vhp-1(km20); Ex[vhp-1p::VHP-1::GFP])* were a gift from the Matsumoto lab. The following strains were obtained from the CGC: CF512 *(rrf-3(b26) II; fem-1(hc17) IV.), KB3 (kgb-1(um3) IV), JT366 (vhp-1(sa366) II), RB2194 (math-33(ok2974) V*, BZ555 *(egIs1 [dat-1p::GFP]), LC108 (uIs69 [(pCFJ90) myo-2p::mCherry* + *unc-119p::sid-1])*, TU3401 *(sid-1(pk3321) V; uIs69 [pCFJ90 (myo-2p::mCherry)* *+* *unc-119p::sid-1])*, and CZ1632 *(juIs76 [unc-25p::GFP* + *lin-15(+)] II)*.

The following strains were produced for this study: PMD13 *(rrf-3(b26) II; egIs1 [dat-1p::GFP])* was made by crossing CF512 with BZ555, PMD14 *(rrf-3(b26) II; fem-1(hc17) IV; juIs76 [unc-25p::GFP* + *lin-15(+)] II)* by crossing CF512 with CZ1632; PMD60 *(rrf-3(b26) II; egIs1 [dat-1p::GFP]; uIs69 [(pCFJ90) myo-2p::mCherry* + *unc-119p::sid-1])* by crossing PMD13 with LC108, PMD63 *(rrf-3(b26) II; egIs1 [dat-1p::GFP]; sid-1(pk3321) V; uIs69 [pCFJ90 (myo-2p::mCherry) + unc-119p::sid-1])* by crossing PMD13 with TU3401. PMD152 *(sid-1(qt9) V; egIs1 [dat-1p::GFP]; uthIs237[myo-3p::tomato, myo-3p::sid-1])* by crossing MAH729 with PMD13; PMD153 *(vhp-1(sa366) II; egIs1 [dat-1p::GFP])* by crossing PMD13 with JT366; PMD62 *(kgb-1(um3) IV; egIs1 [dat-1p::GFP])* by crossing BZ555 with KB3; PMD173 *(math-33(ok2974); egIs1 [dat-1p::GFP])* by crossing BZ555 with RB2194 and PMD112 *(rrf-3(b26); egIs1 [dat-1p::GFP]; utsEx11 [rgef-1p::FLAG::VHP-1::mCherry* + *Cbr-unc-119(+)]* by crossing PMD13 with PMD107.

The *sid-1(pk3321)* mutation was confirmed by resistance to growth arresting RNAi (*act-*5 for neuronal and *pat-10* for body-wall muscle) and by ApoI digestion following single worm PCR with *sid-1(pk3321)* forward and reverse complement (FWD and REV) genotyping primers (Supplementary Table 3).

PMD101 *(utsEx9[rgef-1p::FLAG::PMK-1::mCherry::unc-54 3’ UTR* + *unc-119(+)]; unc-119(ed3) III)*, PMD106 *(utsEx10[sur-5p::FLAG::PMK-1::mCherry::unc-54 3’ UTR* + *unc-119(+)]; unc-119(ed3) III)*, PMD107 *(utsEx11[rgef-1p::FLAG::VHP-1::mCherry::unc-54 3’ UTR* + *unc-119(+)]; unc-119(ed3))*, and PMD117 *(utsEx13[sur-5p::FLAG::VHP-1::mCherry::unc-54 3’ UTR* + *unc-119(+)]; unc-119(ed3))* were generated through plasmid microinjection (see microinjection section).

### RNAi administration

Interfering RNA was obtained from either the Vidal^88^ or Ahringer^89^ RNAi libraries. RNAi targeting the 3’ untranslated region of *vhp-1* mRNA (*vhp-1* 3’UTR RNAi) was designed to 250 base pairs immediately downstream of the *vhp-1* translated region (Supplementary Table 3). All RNAi experiments were performed using HT115 *E. coli* containing either the L4440 empty vector (EV) as a control, or different RNAi constructs. Liquid cultures of RNAi were grown in Terrific Broth (TB) for 14 h at 37 °C and then induced with 1 mM IPTG for 4 h at 37 °C. Cultures were concentrated to 1/10 original volume by centrifugation at 4000 × *g* for 10 min, seeded onto RNAi plates (60 mm or 100 mm, containing 0.1 mg/ml carbenicillin and 1 mM IPTG), and allowed to dry for 1–2 days at room temperature, protected from light. For RNAi that induces larval arrest, *C. elegans* were grown from hatch on EV RNAi until early L4, at which point they were rinsed twice in M9 buffer via centrifugation on a tabletop centrifuge at 1000 × *g* for 15 s, and then transferred to the appropriate RNAi.

### Worm trauma

Age-synchronized *C. elegans* were grown on EV or respective RNAi until day 1 of adulthood. Temperature-restrictive strains were grown at 25 °C to avoid the generation of progeny. Worms were rinsed off growth plates in M9 buffer and pelleted by centrifugation at 1000 × *g* for 15 s. One hundred microliters of worm pellets were then transferred by glass pipette to 2 ml Precellys tubes (Cat. No. 02-682-556, Thermo Fisher Scientific) in a total volume of 500 µl M9. Worms were allowed to settle for ~30 s before loading the tube(s) onto a Precellys Evolution (Bertin Corp, Rockville, MD). The worms were then agitated with the following settings: volume 2 ml, time 16 s, and frequency 6500–10,000 rpm. The range of agitation frequencies was utilized to define non-paralytic, paralytic, and lethal trauma. Worms displayed a paralytic but non-lethal response to agitation between 8600 and 9000 rpm, and this frequency was utilized for all experiments unless noted. Following agitation, worms were pelleted at 1000 × *g* for 15 s and transferred by glass pipette to recovery plates containing a lawn of the respective RNAi or EV on which they were grown. As a control, non-injured worms were likewise suspended in M9 for comparable lengths of time and then transferred to recovery plates without having received trauma.

### Paralysis assay

As described above, CF512 worms were injured at either 6500, 7500, 8600, 9000, or 10,000 rpm. Immediately following injury for three biological replicates, worms were transferred in M9 with a glass pipette to recovery plates in droplets containing 30–100 worms each. The number of paralyzed and non-paralyzed worms were counted in one or two droplets for each condition. Worms were scored as paralyzed if they failed to exhibit movement by thrashing in M9.

### Worm death assay

CF512 animals were injured at 6500, 7500, 8600, 9000, or 10,000 rpm and placed on recovery plates for 48 h. Worms were then rinsed off plates with M9 buffer, washed twice with fresh M9 buffer via 1000 × *g* centrifugation at room temperature for 30 s, and then stained with 1 μg/ml propidium iodide protected from light for 10 min with agitation. Following staining, worms were washed an additional time with M9 buffer, centrifuged (1000 × *g*) for 1 min at room temperature and then analyzed by large-particle flow cytometry, utilizing a 561 nm laser to excite propidium iodide incorporated into dead worms.

### Microscopy and neurodegeneration scoring

Non-injured control worms and injured worms were imaged 1 and 48 h post injury. Worms were mounted in M9 supplemented with 100 mM NaN_3_ on glass slides and coverslipped. Dopaminergic neurons and muscle cells were imaged on a Leica SP8 confocal microscope (Leica, Buffalo Grove, IL) with a ×20 objective under water immersion. PMD14 worms expressing GFP in GABAergic neurons were imaged with a ×10 objective with air immersion. Scoring dopaminergic neurons for degeneration was adapted from previous methods^90,91^. PMD13 worms expressing GFP in dopaminergic neurons were scored for the number of healthy or degenerate CEP neurons by manual counting after performing Z-stacks comprising the entire height of the worm head and obtaining a maximum projection of that Z-stack (LASX Software, Leica). Abnormal CEP neurons were counted when cell bodies were missing or fragmented, or when dendrites exhibited beading or breakage. To display the relative GFP fluorescence intensity of control or injured worms, Z-stacks were flattened by average projection (LASX Software). Image projections were analyzed by ImageJ (NIH, Bethesda, MD) to determine the peak GFP signal. For GABAergic neurons, maximum projections of confocal Z-stacks were analyzed to count the number of normal or abnormal GABA commissures along the body of the worm. Commissures were counted as abnormal if they exhibited breaks or beading. AGD1651 worms expressing myo-3p::LifeAct::mRuby to label muscular actin filaments were imaged in a single plane by confocal microscopy. Quantification was performed on the number of worms containing any signs of damaged muscle architecture (broken actin filaments) compared to normal architecture.

### Dye-filling

DiI (1,1′-Dioctadecyl-3,3,3′,3′-Tetramethylindocarbocyanine Perchlorate, cat. no. D3911, Thermo Fisher) was dissolved in dimethyl sulfoxide (DMSO) to a 20 mg/ml stock solution. CF512 worms were washed off plates with M9 buffer, then rinsed once with M9 buffer (1000 x g at room temperature for 30 s) and placed into 500 µl M9 with 0.01% Triton-X 100 and 1:200 DiI stock in a single well of a 9-well glass plate. Plates were protected from light and incubated for 1 h at room temperature with gentle rocking. Following incubation, worms were transferred to plates containing a fresh lawn of OP50 and further incubated for 1 h at room temperature. Worms were then rinsed off plates with M9 buffer, washed twice with fresh M9 buffer (1000 × *g* at room temperature for 30 s), and anesthetized with 250 mM Sodium Azide on NGM plates. DiI fluorescence was imaged using a Zeiss Axio Zoom.V16 (Zeiss, Oberkochen, Germany) with a Texas Red filter.

### Large-particle flow cytometry

Age-synchronized worms were analyzed by flow cytometry with a COPAS FP-250 flow cytometer (Union Biometrica, Holliston, MA) using the attached sample cup or acquired from a 96-well plate by an LP Sampler (Union Biometrica). The sample solution was comprised of M9 while the flow sheath solution contained a proprietary recipe, COPAS GP SHEATH REAGENT (PN: 300-5070-100, Union Biometrica). Flow data were collected and processed using the FlowPilot software (ver. 2.6.1, Union Biometrica). Further data processing was performed in Excel (Microsoft) and statistical analysis in Prism (Graphpad, San Diego, CA). Gating strategies to remove larvae, *E. coli*, worm doublets, and curled up worms are described in Supplementary Fig. 8. Extinction was detected using the 488 nm laser line with a 1.3ND filter and gain of 1.0. Propidium iodide and mCherry were excited using a 561 nm laser while GFP was excited with the 488 nm laser. Gains for fluorescence detection were set to 2.0 and PMT voltage adjusted within the linear range of the instrument and was consistent for each experiment.

### Manganese treatment

PMD13 worms were grown on OP50 bacteria at 25 °C until L3/L4 and then rinsed off plates with M9 buffer, washed once with fresh M9 buffer (1000 × *g* at room temperature for 30 s) and treated with either 50 mM Sodium Chloride solution (0 mM MnCl_2_) or 50 mM Manganese Chloride solution (50 mM MnCl_2_) for 1 h with agitation. Following incubation, worms were rinsed with M9 buffer three times (1000 × *g* at room temperature for 30 s) and placed upon recovery NGM plates containing OP50 bacteria. Worms were analyzed for dopaminergic GFP fluorescence 24 h later by large-particle flow cytometry.

### Behavioral assays

For gentle nose-touch assays, CF512 worms were grown on EV or *vhp-1* RNAi plates until day 1 of adulthood, at which point half of each condition were given paralytic trauma and the other half maintained as non-injured controls. Beginning 24 h post trauma and continuing every 24 h until day 4 post trauma, non-injured control and injured worms were assayed for nose-touch response, in which an eyelash was gently brushed along a side of the worm nose. A positive response was scored when the worm stopped and performed a reversal in movement. This procedure was repeated five times for each worm scored, and each worm was given a value representing the percent of positive responses to nose touch. The nose-touch assay was only performed on injured worms which clearly exhibited the ability to move.

Chemotaxis assays were performed on 60 mm chemotaxis plates (1.6% agarose, 5 mM KPO_4_, pH 6.0, 1 mM MgSO_4_, and 1 mM CaCl_2_), which were prepared by delineating four quadrants on each plate with a central 1 cm circle. A 2 µl drop of 1 M sodium azide was added near the center-edge of each quadrant, followed by a 2 µl drop of 10% butanol in ethanol (chemoattractant) on two opposite quadrants, or a 2 µl drop of ethanol (control) in the other two quadrants. Injured or non-injured CF512 worms (~100) were placed in the 1 cm diameter circle in the middle of the 60 mm chemotaxis plates. Worms were allowed to chemotax for 1 h at 20 °C, after which point the number of worms that had moved to each quadrant was counted and the chemotactic index calculated according to the formula:

1 $${\mathrm{Chemotactic}}\;{\mathrm{Index}} = \frac{{\left( {\# \mathrm{butanol}} \right) - (\# {\mathrm{ethanol}})}}{{(\# {\mathrm{total}})}}$$

To assess worm movement, non-injured control or injured CF512 worms were assayed for movement 48 h after injury. Single worms were gently picked off recovery plates with a worm pick and placed on fresh plates with a lawn of OP50 bacteria. Worms were allowed to move freely for 60 s, after which time their movement phenotype was manually scored.

### Reverse genetic screening

Genes significantly upregulated by microarray in day 1 adult injured animals were filtered based on expression threshold >1, expression in mechanosensory neurons, availability in the Vidal RNAi library, and human homology by BLAST. These genes were knocked down by RNAi in PMD13 worms, and the worms were subjected to moderate trauma as described above. Dopaminergic GFP fluorescence was scored 48 h later by large-particle flow cytometry. The relative loss of GFP signal in injured versus uninjured animals was calculated for each RNAi condition and compared to the signal lost in injured animals raised on EV bacteria during that experiment. This value was termed the “Dopaminergic GFP Index” and is given by the formula:

2 $${\mathrm{GFP}}\,{\mathrm{Index}} = \frac{{({\mathrm{EV}} - {\mathrm{RNA}}i)}}{{({\mathrm{EV}} - 1)}}$$

where EV represents the GFP fluorescence in the population of injured over uninjured worms for the empty vector condition used as a control for each independent experiment. “RNAi” likewise represents the retained GFP fluorescence from the proper RNAi condition.

### Western blotting

Unless noted, western blot analyses were performed on Day 1 adult animals. Age-synchronized worms were rinsed in M9 buffer, pelleted by centrifugation at 1000 × *g* at room temperature for 30 s, washed once with M9 buffer (1000 × *g* at room temperature for 30 s), and transferred in minimal volume to a 1.5 ml microcentrifuge tube. Worm pellets were flash frozen in liquid nitrogen and stored at −80 °C until processed. Worm extracts were generated by glass and zirconia bead-beating in the presence of denaturing lysis buffer (50 mM HEPES pH 7.4, 150 mM NaCl, 1 mM EDTA, 0.5% SDS, 1% Triton-X 100, 1X EDTA-free protease inhibitor cocktail (Roche), and 1× phosSTOP phosphatase inhibitor cocktail (Roche). Raw lysates were centrifuged at 10,000 × *g* for 5 min at 4 °C and supernatants were transferred to a fresh tube. Protein concentration was determined by BCA protein quantification kit (Thermo Scientific). All protein samples for an experiment were adjusted to an equivalent concentration by the addition of denaturing lysis buffer. Lysates were supplemented 1:1 with 2× SDS sample buffer and boiled at 90 °C for 10 min, resolved by bis/acrylamide SDS-PAGE electrophoresis, transferred to nitrocellulose membranes, and subjected to analysis by western blot. Band intensities were quantified by ImageJ (NIH) or Image Studio Lite (ver. 5.2, Licor, Lincoln, NE) and normalized to tubulin. Filter trap analysis was performed on worm extracts supplemented to a final concentration of 0.1% SDS and allowed to filter through equilibrated cellular acetate membranes via BioRad slot-blot apparatus by gravity. Wells were allowed to dry before immunoblotting with anti-GFP antibodies and visualized by Licor.

### Co-immunoprecipitation

Frozen worm pellets were lysed by glass/zirconia bead beating in the presence of CHAPS lysis buffer (50 mM HEPES pH 7.4, 100 mM NaCl, 1 mM EDTA, 1% CHAPS, and 1× EDTA-free protease inhibitor cocktail (Roche)). Lysate protein concentrations were determined by BCA protein quantification kit. 2 mg/ml of protein was added to 1 µg primary antibody in 400 µl total volume of CHAPS lysis buffer and incubated with rotation at 4 °C for 1 h. VHP-1::GFP was immunoprecipitated with mouse anti-GFP monoclonal antibody (Cat. No. 11814460001, Roche). A portion of raw lysate was saved for analysis of total protein input. Following incubation, 100 µl Protein A or G magnetic bead (Surebeads, Cat. No. 1614013 and 1614023, Bio-Rad, Hercules, CA) slurry washed four times with ice-cold native lysis buffer (50 mM HEPES pH 7.4, 100 mM NaCl, 1 mM EDTA, 1% Triton-X 100) was added to each sample, and samples were incubated for an additional hour at 4 °C with rotation. The beads were then pelleted by magnet and washed five times with ice-cold native lysis buffer. Proteins were eluted by addition of 1X sample buffer and boiling at 90 °C for 10 min.

### Quantitative PCR

Quantitative, reverse-transcriptase PCR (qPCR) was performed on day 1 adult CF512, KB3, and PMD60 worm strains. Worms were age-synchronized by hypochlorite treatment and grown on respective RNAi or EV at 25 °C until D1 of adulthood. Worms were washed off plates with M9 buffer and collected by centrifugation at 1000 × g for 30 s at room temperature prior to receiving either moderate trauma (treatment group) or no trauma (control group). The treatment group was recovered for 1 h at 20 °C on a lawn of appropriate RNAi. Worms were harvested in TRIzol (ThermoFisher Scientific) flash-frozen in liquid nitrogen and retained at −80 °C until processing. Total RNA was extracted by freeze-thawing three times with vortexing followed by a chloroform/phenol and isopropanol precipitation extraction process. RNA pellets were rinsed twice with 75% ethanol, air-dried, and re-suspended in 20–50 µl molecular biology grade water. A DeNovix spectrometer (DS-11 FX+, DeNovix, Wilmington, DE) was used to measure RNA concentration, 260/280 ratios, and 260/230 ratios. Utilizing the QuantiTect Reverse Transcription kit (Qiagen), 1 µg of RNA was used to synthesize cDNA following the manufacturer’s instructions, including the gDNA wipeout step. For qPCR, 20 µl reactions were performed using the iTaq^TM^ Universal SYBR® Green Supermix kit (Bio-Rad) with 5 ng cDNA per well in a CFX384 Real-Time System (Bio-Rad). Each sample was loaded in technical triplicate, and at least three biological replicates were analyzed for each condition. Fidelity of PCR reactions was examined by melting curve analysis. Melting curves with >1 significant peak were excluded from technical triplicates during analysis. The relative transcript levels for each target gene and two housekeeping genes, *tba-1* and Y45F10D.4, were calculated with the ∆Ct method. The geometric mean of the two housekeeping transcripts were used to normalize target gene expression. Forward (FWD) and reverse complement (REV) primers are listed in Supplementary Table 3.

### Closed head traumatic brain injury

Group-housed, B6D2F1 mice at 8–10 weeks of age at 21–23 °C and 35–70% humidity were allowed access to food and water *ad libitum* and treated in accordance with approved IACUC protocols. The closed-head traumatic brain injury device consisted of an upright, railed-guided weight drop composed of an aluminum body and equipped with a speed monitoring device to measure the impact velocity of the weight drop. The modular weight system consisted of a 50 ml polypropylene conical tube with lead ball bearings at 220 g. The weighted conical tube lands on the brass impactor containing a slightly concave nylon tip which is in direct contact with the mouse head and centered along the sagittal suture of the skull. Before injury, mice were anesthetized (2% isofluorane) and placed on a thick memory foam cushion on a height-adjustable platform. The weight is dropped by activating an electronic trigger followed by a 1 m drop. After recovery, mice were placed back in their cage with food and water *ad libitum*. Control mice received only anesthesia and were not subjected to impact.

### Cloning

All plasmids generated for this study were assembled utilizing a Gibson Assembly Kit (Cat. No. E5510S, NEB, Ipswich, MA) following the manufacturer’s recommended protocol utilizing 0.02 pmol vector and 0.06 pmol of each insert. *Vhp-1* was cloned from *C. elegans* cDNA and subsequently cloned into linearized pNB1 and pNB20 (provided by Dr. Andrew Dillin) plasmid vectors with mCherry cloned from pRL2980 (med-1p::mCherry::mex-3, provided by Dr. Rueyling Lin). Primers containing 15–20 nucleotide homologous overhangs were utilized to amplify the vector backbone and fragments for assembly. Vectors and fragments were amplified using Q5 High-fidelity polymerase (Cat. No. M0492S, NEB) master mix, and PCR settings: initial denaturing 95 °C, 30 s (hot start), 95 °C for 10 s, 60 °C annealings for 30 s, and 72 °C extensions for 30–240 s, repeated for 2 cycles. Followed by: 95 °C for 10 s, 68–72 °C annealing for 30 s, and 72 °C extensions for 30–240 s, 72 °C for 5 min final extension, repeated for 33 cycles. Following PCR, products were loaded onto a 0.8% agarose/TBE gel and electrophoresis was performed at 120 V for 1–2 h. Bands of interest were excised from the gel and gel purified using a Zymoclean Gel DNA Recovery Kit (Cat. No. D4007, Zymo Research, Irvine, CA) following the manufacturer’s instructions. The concentration of gel-purified DNA was measured with a DeNovix DS-11 FX+.

### Worm microinjections

Worms were injected using a FemtoJet 4i (Eppendorf, Hamburg, Germany) attached to a Leica DMi8 inverted stereomicroscope (Leica) using 0.78 mm ID/1.0 mm OD pulled glass capillaries. Injection solutions contained 100 ng/µl of plasmid in molecular biology grade water. For injections with multiple plasmids, the injection mixture contained 100 ng/µl of each plasmid. Injection solutions were loaded into glass needles by the capillary effect and needle tips were broken by gently tapping against the edge of a glass coverslip. Young adult worms were mounted in Halocarbon 700 oil (Cat. No. H8898, Sigma Aldrich, St. Louis, MO) and injected once in each arm of the gonad. Injection efficiency was monitored by visualization of the gonad expanding upon injection. The animals were then suspended in M9 and recovered onto a fresh lawn of OP50.

### Microarray

CF512 worms were grown to either Day 1 or Day 4 of adulthood and given moderate injury on a Precellys 24 (Bertin) at max settings for 20 s. Samples were collected 1 h post injury in biological triplicates. Raw expression data were obtained with the Affymetrix *C. elegans* Genome Array. Total RNA was purified from worms by phenol/chloroform extraction followed by isolation with the RNeasy mini kit (Qiagen). Preprocessing of raw expression data was performed using Bioconductor^92^ and the GC-RMA method^93^. Processing included multi-chip averaging, background adjustment, and quantile normalization. Analysis of gene regulation was performed in MATLAB. Genes were considered significantly regulated by injury when fold change was ≥2.0 and *p*-value ≤ 0.05 (unpaired *t*-test). Significantly regulated genes by trauma were compared to other published transcriptional stress responses [accession numbers: GDS4568^58^, GDS1379^94^, GDS4570^95^, GSE91073^96^, and GSE100814^97^] by cross-correlation analysis in MATLAB. Also compared was heat shock transcription from ref. ^98^. PANTHER (http://pantherdb.org/) and the WormBase Enrichment Analysis tool (https://wormbase.org/tools/enrichment/tea/tea.cgi) were utilized for GO-term enrichment analysis. Enriched terms with False Discovery Rate (FDR) corrected *p*-value of ≤0.1 were considered significant. Raw data deposited in NCBI Geo Datasets (GSE148325, ID 200148325).

### Cross-correlation analysis

Each *C. elegans* dataset was filtered for genes significantly regulated in our worm trauma dataset. These expression data (as fold-change values) were loaded into MATLAB using the “importdata” function. Each dataset was then normalized by subtracting the mean and dividing by standard deviation. Following normalization, NaN values were removed from datasets using a custom MATLAB function incorporating the MATLAB “isnan” function and each individual dataset filtered and concatenated such that expression data from each gene contained the same index in each dataset. To generate a cross-correlation matrix, all normalized expression datasets were used as column inputs to the “corrcoef” function, giving the cross-correlation values.

### Illumina RNA sequencing (RNAseq)

Three biological replicates of day 1 adult PMD60 worms were collected for each of the following conditions: EV uninjured, EV injured, *vhp-1* RNAi uninjured, and *vhp-1* RNAi injured. Samples were collected one hour after injury. RNA was purified by chloroform/phenol extraction followed by isopropanol precipitation and two washes with 75% ethanol before resuspension in 50 µl molecular biology grade water. Quality control, mRNA purification, and paired-end 150 bp Illumina sequencing was performed by Novogene (Sacramento, CA). Briefly, mRNA was enriched using oligo(dT) beads, randomly fragmented in fragmentation buffer, and reverse transcribed to cDNA using random hexamers. Following first-strand synthesis, Illumina synthesis buffer was added with dNTPs, RNase H, and *E. coli* polymerase I to synthesize the second strand by nick-translation. The cDNA library was then purified, underwent terminal repair, A-tailing, and ligation of adapters before PCR enrichment. Before Illumina sequencing, the cDNA library concentration was quantified with a Qubit 2.0 fluorometer (Thermo Fisher) and sized with an Agilent 2100 Bioanalyzer (Agilent, Santa Clara, CA). Following sequencing, error rates were <0.03% for each sample and contained >99% clean reads when accounting for adapter related reads. Greater than 99% of all reads mapped to exons. RNAseq statistical analysis was performed using CLC software (version 9.5, CLC Bio, Aarhus, Denmark). Gene expression data were compared by Baggerly’s test with False Discovery Rate (FDR) correction. Genes were considered significantly regulated by trauma or *vhp-1* RNAi when absolute fold-change ≥2 and FDR *p*-value ≤ 0.05. Raw data deposited in NCBI Geo Datasets (GSE148337, ID 200148337).

### Short linear motif analysis

The VHP-1 protein sequence was analyzed using the APC/C Degron repository (http://slim.icr.ac.uk/apc/index.php) and the Eukaryotic Linear Motif (ELM) prediction server (http://elm.eu.org/) with a motif probability cutoff of 100. Statistically significant findings for degrons (black lines) or binding sites for components of the ubiquitin-proteasome pathway (red) were aligned with the full-length sequence of VHP-1.

### MG132 treatment

RNAi plates containing a lawn of EV bacteria were supplemented with either DMSO (vehicle) or DMSO + sufficient MG132 (Cat. No. S2619, Selleckchem, Houston, TX) to a final 50 µM working agar concentration. Following absorption of DMSO/MG132 into plates, day 1 adult KM20 worms were transferred in M9 buffer by glass pipette to prepared plates and incubated at 20 °C. At the indicated time-points, worms were washed from plates with M9 and snap-frozen in liquid nitrogen.

### Statistical analysis

All statistical analysis was performed using Prism 8 software (GraphPad) unless noted. Post-hoc analysis was determined by Prism recommendation. Reported are corrected *p*-values as follows: *p = 0.05–0.01, **p = 0.01–0.001, ***p = 0.001–0.0001, ****p < 0.0001. Statistical analysis of large-particle flow cytometry data was performed in both Prism and Excel. To evaluate large worm numbers across biological replicates, error propagation was performed according to the general formula:

3 $$\delta R = \sqrt {\left(\frac{{\partial R}}{{\partial X}} \ast \delta X\right)^2 + \left(\frac{{\partial R}}{{\partial Y}} \ast \delta Y\right)^2 + \ldots }$$

where *δR*, the total error within each independent repeat, is a function of each independent variable (*X, Y* …). For the particular case of error propagation for the dopaminergic GFP Index within each of multiple biological repeats, error is further added in quadrature and the function becomes:

4 $$\delta R = \sqrt {\mathop {\sum}\limits_X {\left(\frac{{\delta X}}{{1 - \mathrm{EV}}}\right)^2} }$$

where *δX* represents the error and *X* contains all biological replicates. EV is a constant determined by the relative loss of fluorescence observed in injured worms grown on EV bacteria during that experiment. Lastly, the total error within each repeat was added to the error between replicates in quadrature.

### Homology analysis

Worm genes were examined for annotated mammalian homologs in Wormbase (version: WS278). In the absence of annotated homologs, the amino acid sequences for worm gene products were examined in UniProt with BlastP against uniprotKB_mammals database using the blosum62 matrix with an E-Threshold = 10, matrix = Auto, and no filtering.

### Statistical analysis

Statistical analyses, including *t*-test, ANOVA, Chi-squared, and mixed-effects analysis, were conducted using Prism (version 8.4) and CLC genomics workbench (version 9.5).

### Reporting summary

Further information on research design is available in the Nature Research Reporting Summary linked to this article.

## Supplementary information

Supplementary Information

Description of Additional Supplementary Files

Supplementary Data 1

Supplementary Data 2

Supplementary Data 3

Supplementary Data 4

Supplementary Data 5

Supplementary Data 6

Reporting Summary

## Data Availability

All data generated and analyzed during this study are included in this article and its Supplementary Information and are also available from the authors upon reasonable request. Transcriptomic data files that support the findings of this study on mechanical stress in *C. elegans* have been deposited in the NCBI Gene Expression Omnibus (GEO). Illumina HiSeq 2500 datasets were assigned an accession code of GSE148337 and an identifier of 200148337. Affymetrix *C. elegans* Genome microarrays were assigned an accession code of GSE148325 and an identifier of 200148325. Source data are provided with this paper.

## Source data

Source Data
