# Supplementary material for: Trauma-induced regulation of VHP-1 modulates the cellular response to mechanical stress
Source: Nat Commun. 2021 Mar 5;12:1484. doi: 10.1038/s41467-021-21611-8 (PMC7935884; doi:10.1038/s41467-021-21611-8)
Supplement: Supplementary file 1 — Supplementary Information [file 41467_2021_21611_MOESM1_ESM.pdf]

Supplementary Information:

Trauma-induced regulation of VHP-1 modulates the cellular response to mechanical stress

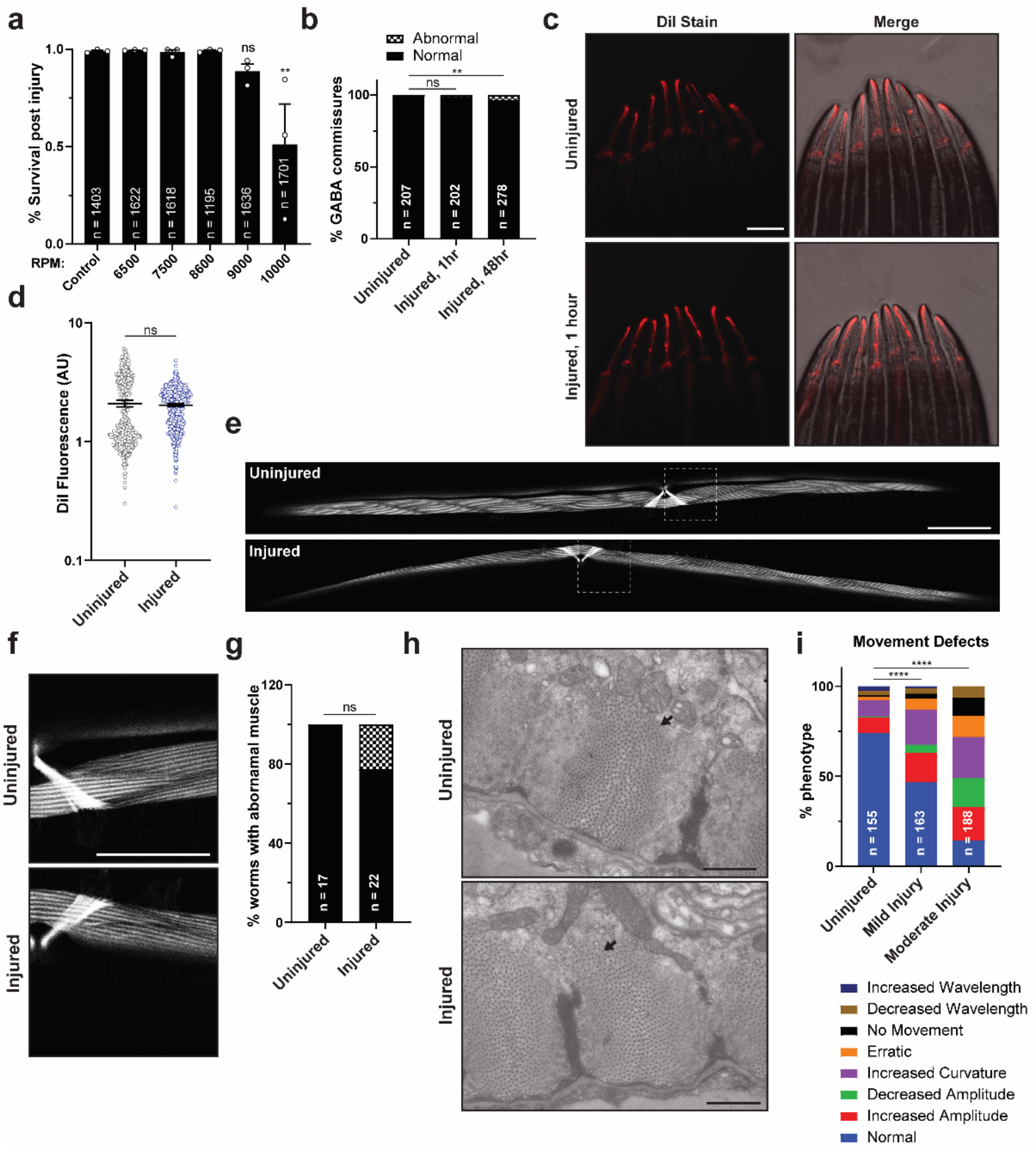

Supplementary Figure 1. Effects of blunt force trauma on worm morphology and movement. (**a**) Survival of worms (CF512) in response to injury at increasing frequencies for 16 sec. Worms were analyzed by propidium iodide staining and flow cytometry. Control is Uninjured. Mean + SEM, ns (not significant), **p = 0.0084 by one-way ANOVA with Dunnett test, n value for animal number reported on bar graph from 3 independent trials. (**b**) Quantification of abnormal GABAergic commissures with or without injury from confocal micrographs (Fig. 1d). ns (not significant), **p = 0.0058, chi-squared test. n = number commissures counted. Black (normal), checkered (abnormal). (**c**) Dye-filling assay using DiI to stain head neurons. Shown are fluorescence micrographs (left) and merge with phase contrast (right). Reference Supplementary Fig. 1d. Scale = 100 µm. (**d**) Quantification of dye-filling assay by large-particle flow cytometry. Mean ± 95% CI, ns (not significant) p = 0.1678, two-sided t-test. n = 417 (2 trials), Uninjured, n = 645 (2 trials), Injured. Arbitrary units, AU. (**e,f**) Confocal micrographs of transgenic worms expressing *myo-3p::LifeAct::mRuby* (AGD1651) imaged within 1 hour (hr) of trauma. Scale = 100 µm. Dashed box indicates zoom regions. (**f**) Zoom of (e) showing fluorescently marked actin filaments. Reference Supplementary Fig. 1g. Scale = 50 µm. (**g**) Quantification of muscle damage seen in worms after injury. ns p = 0.0565, chi-squared test. Black (normal), checkered (abnormal). (**h**) TEM micrographs depicting *C. elegans* muscle cells in uninjured or injured worms (CF512). Arrows indicate thick filaments. n = 3 independent experiments. Scale = 500 nm. (**i**) Quantification of movement defects observed in worms after mild (7500 rpm) or moderate injury (8600 rpm). ****p < 0.0001, chi-squared test. Color scale below indicates the movement phenotype.

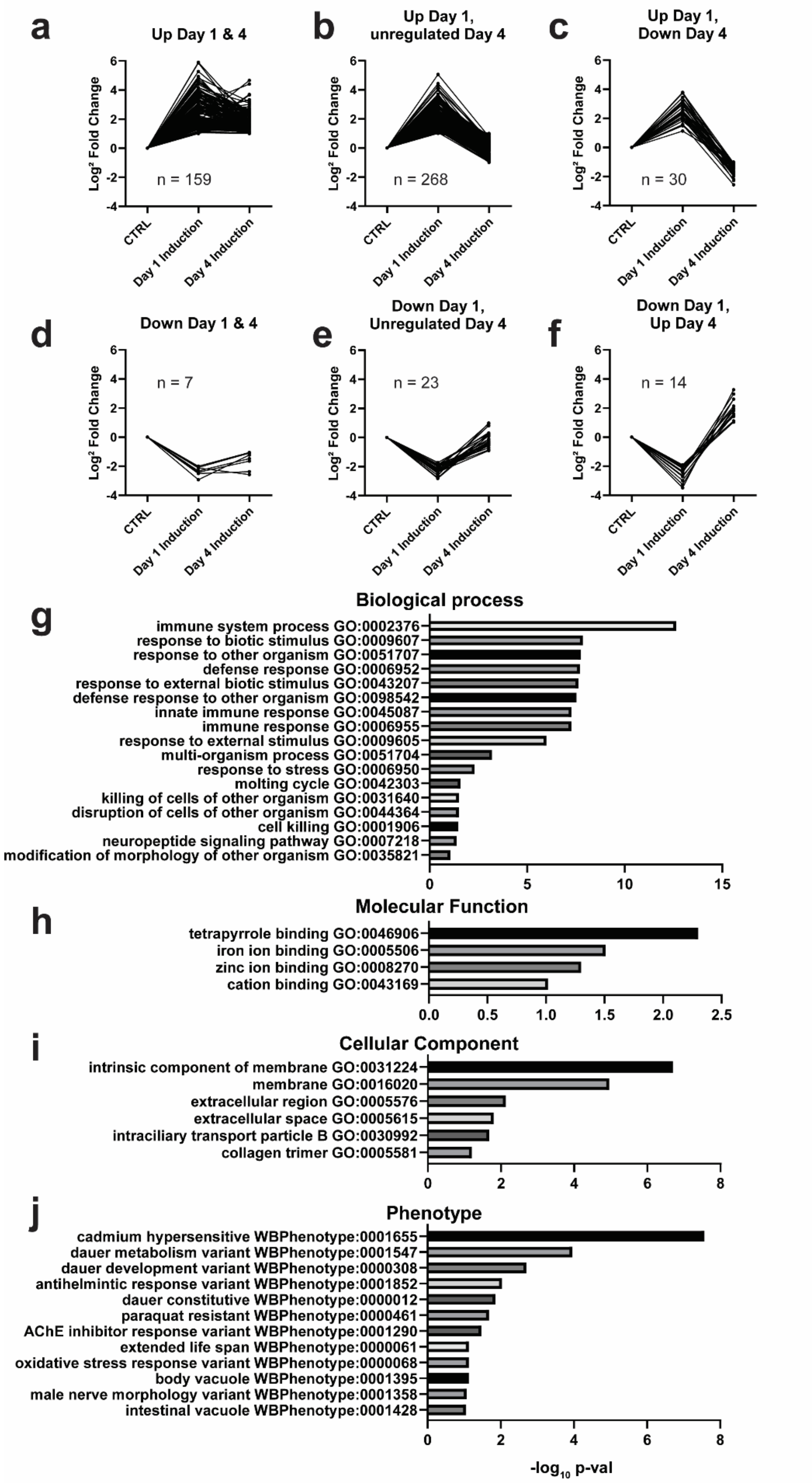

Supplementary Figure 2. Trauma elicits an age-dependent transcriptional response enriched in stress-associated elements. (**a-f**) Comparison of significantly activated genes in Day 1 adult worms versus worms given trauma on Day 4 of adulthood. Genes classified as "regulated" at Day 4 of adulthood if absolute Log_2 fold change ≥ 1 compared to age-matched controls. (**g-j**) GO-term (gene ontology) and phenotype analysis from WormBase and PANTHER databases. Plots display over-represented terms (FDR p-value ≤ 0.10) from genes significantly regulated in Day 1 adult animals.

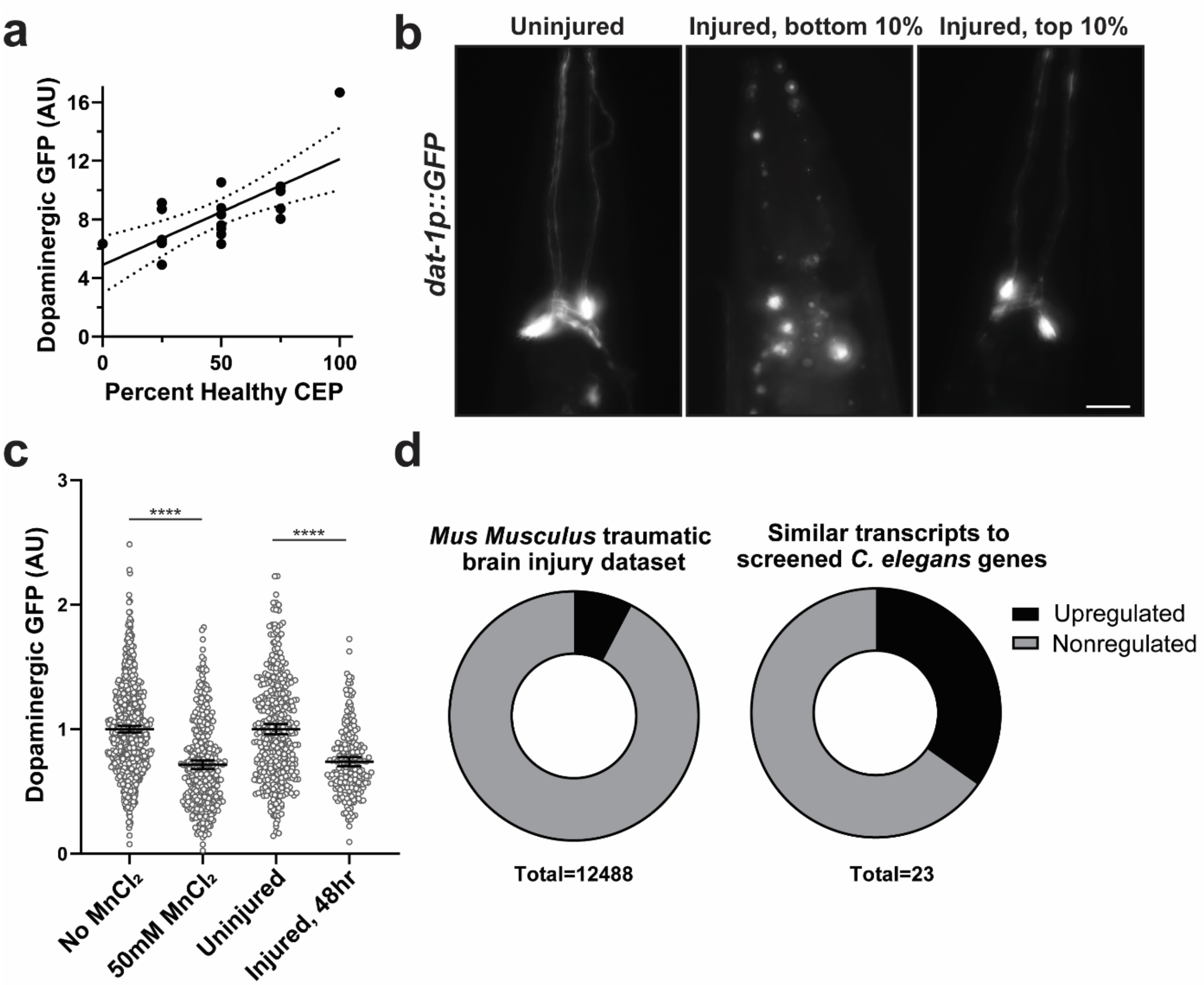

Supplementary Figure 3. Dopaminergic fluorescence by flow cytometry is indicative of neuronal health. (**a**) Correlation between dopaminergic GFP fluorescence and percentage of normal CEP neurons scored from confocal micrographs. Spearman $r = 0.7100$, ***p = 0.0007. Line represents linear regression with 95% CI. (**b**) Fluorescence micrographs following sorting by large particle flow cytometry of transgenic worms (PMD13) expressing GFP in dopaminergic neurons. Injured worms were sorted from two gates representing the bottom and top 10th percentile of GFP fluorescence. n = 3 independent experiments. Scale = 20 µm. (**c**) Large particle flow cytometry analysis of worms (PMD13) subjected to 50 mM $MnCl_2$ treatment or trauma (48 hours, hr, post trauma). ****$p < 0.0001$, two-way ANOVA with Sidak test. See Supplementary Data 5 for additional statistics. (**d**) Enrichment analysis comparing unique mouse homologs/analogs of *C. elegans* genes meeting screening criteria to transcriptomic analysis of mouse traumatic brain injury by lateral CCI, Microarray (GDS2850). Part of the whole indicates genes induced by >1.5-fold by trauma, upregulated (black) versus unaffected genes (gray). ***p = 0.0002, two-sided Fisher's exact test. See Supplementary Data 6.

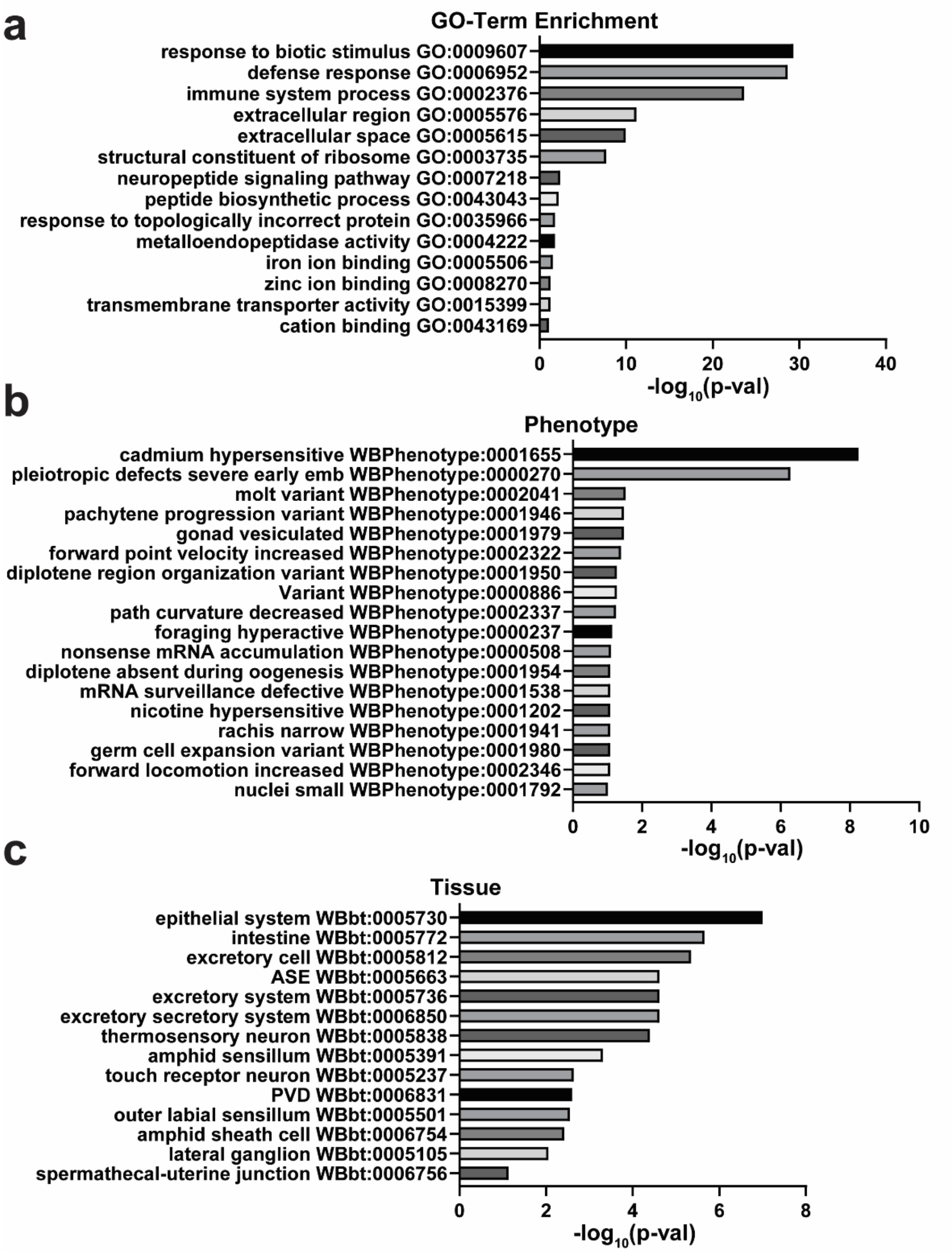

Supplementary Figure 4. Related to Figure 4. Trauma and *vhp-1* RNAi activate shared stress-responsive and neuronal genes. (**a-c**) Significantly over-represented (FDR p-value ≤ 0.10) WormBase GO- (gene ontology), Phenotype-, and Tissue- terms in the 460 genes upregulated under both injury and *vhp-1* RNAi conditions by RNAseq.

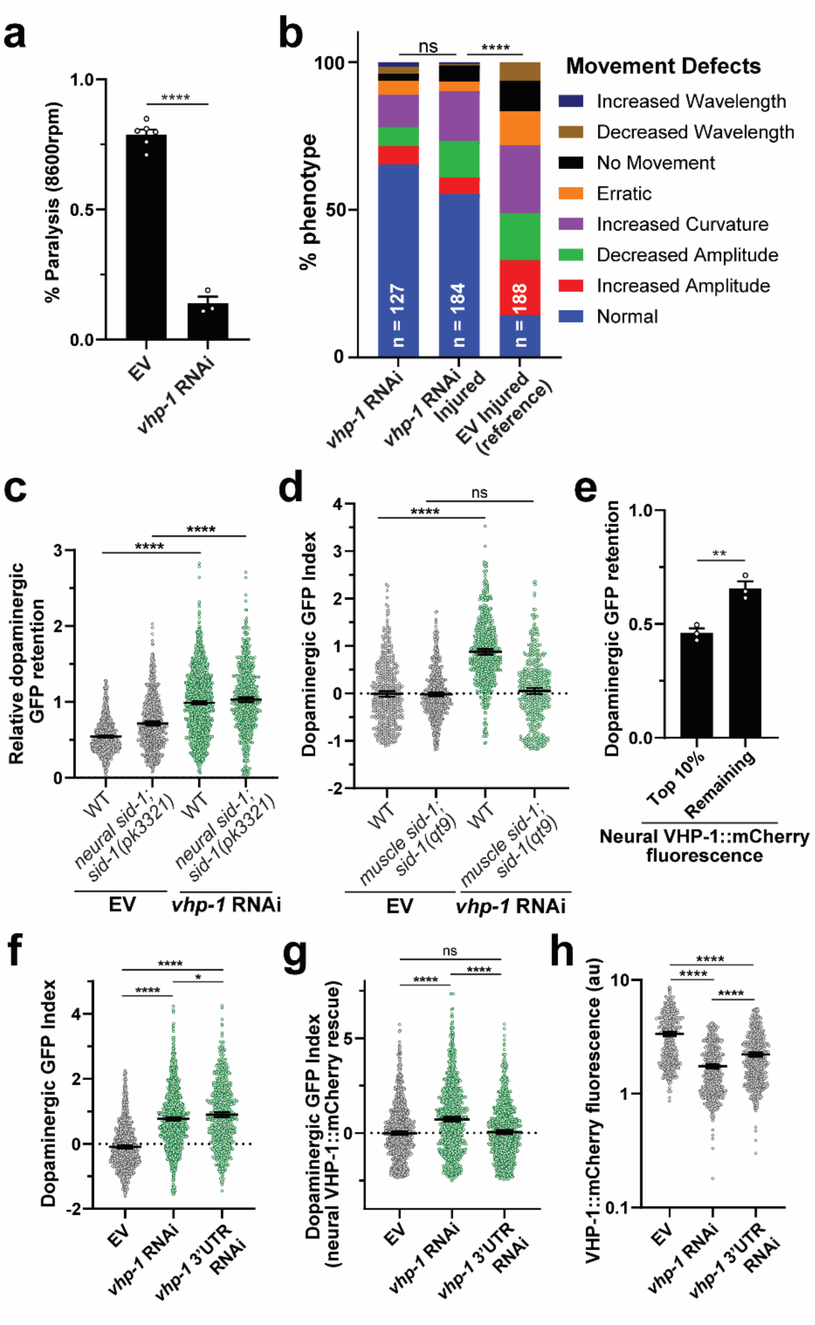

Supplementary Figure 5. Protective effects of *vhp-1* RNAi within the nervous system. (**a**) Percent of injury-induced animal paralysis on control (EV) or *vhp-1* RNAi. Mean ± SEM, ****p < 0.0001 by two-sided unpaired t-test. (**b**) Quantification of movement defects observed in worms after moderate injury (8600 rpm) on *vhp-1* RNAi. Control (EV) injury reference from Supplementary Figure 1i. ****p < 0.0001, ns (not significant), two-sided chi-squared test. Color scale on right indicates movement phenotype. n values represent number of worms over 3 independent trials. (**c,d**) Large-particle flow cytometry results from *vhp-1* RNAi in worms with all-tissue RNAi sensitivity (PMD60, c; PMD13, d) denoted as wild type (WT) or *sid-1* mutant worms sensitive to RNAi in neurons (PMD63, c) or body-wall muscle (PMD152, d) alone. Shown is relative dopaminergic GFP retention, c, and the dopaminergic GFP index, d, at 48 hours post injury. Circles represent single worms on either empty vector (EV, gray) or *vhp-1* RNAi (green). Mean ± 95% CI. Two-way ANOVA with Tukey multiple comparison test. See Supplementary Data 5 for additional statistics. (**e**) Large-particle flow cytometry of transgenic worms expressing VHP-1::mCherry in the nervous system (PMD112). GFP retention in dopaminergic neurons 48 hours after trauma from the top 10% of mCherry fluorescing animals was compared to the remainder of the injured population. Mean + SEM, **p = 0.0052 by two-sided unpaired t-test, n = 3107 over 3 independent trials. (**f,g**) Large-particle flow cytometry results from *vhp-1* RNAi and *vhp-1* 3'UTR RNAi in control (PMD13, f) or transgenic animals expressing neuronal VHP-1::mCherry (PMD112, g). Shown is the dopaminergic GFP index at 48 hours post injury. Circles represent single animals on either empty vector (EV, gray) or *vhp-1* RNAi (green). Mean ± 95% CI. One-way ANOVA with Tukey multiple comparison test. See Supplementary Data 5 for additional statistics. (**h**) Quantification of neuronal VHP-1::mCherry fluorescence in individual animals, gray circles, by large-particle flow cytometry with empty vector (EV), *vhp-1* RNAi and *vhp-1* 3'UTR RNAi. Mean ± 95% CI. One-way ANOVA with Tukey multiple comparison test. See Supplementary Data 5 for additional statistics.

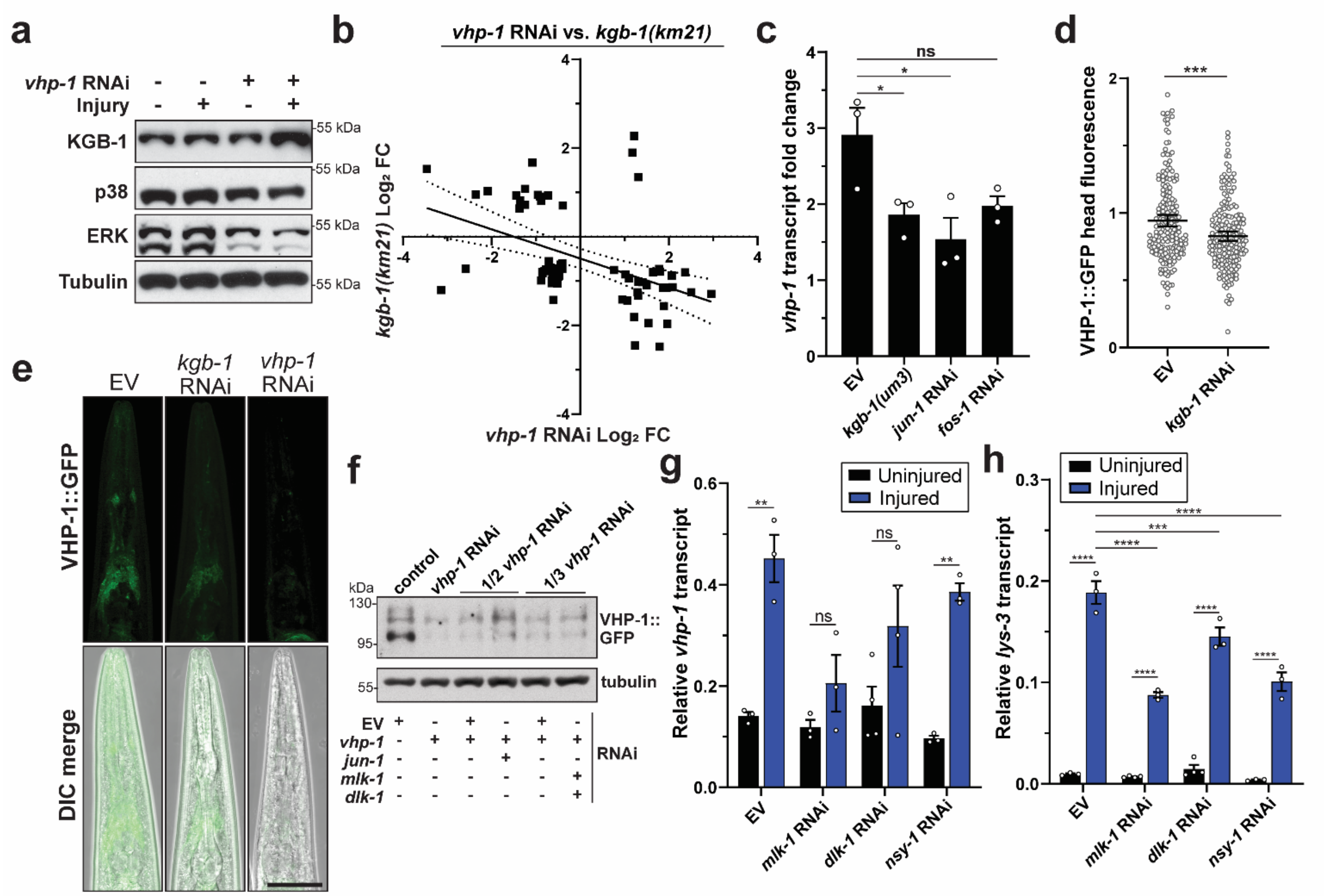

Supplementary Figure 6. Influence of MAPK pathway components on neuroprotection by *vhp-1* RNAi. (**a**) Western blot of total KGB-1, p38, and ERK in response to injury and/or *vhp-1* RNAi. Tubulin, loading control. n = 3 biological replicates. (**b**) Plot comparing fold-change of significantly regulated genes (FDR p-value ≤ 0.05) by RNAseq of worms raised on *vhp-1* RNAi to microarray of *kgb-1(km21)* mutant worms [accession number: GSE82238, NCBI GEO2R top 250 analysis]. Spearman *r* = -0.5816, ****p < 0.0001. Line represents linear regression with 95% CI. FC, fold change. (**c**) qPCR shows fold-change of *vhp-1* transcript after injury with empty vector (EV), *jun-1*, and *fos-1* RNAi as well as *kgb-1(um3)* mutant animals. Mean + SEM, from left to right, *p = 0.0429 and 0.0116, ns (not significant) p = 0.07 by one-way ANOVA with Dunnett test, n = 3 biological replicates. (**d**) Quantification of VHP-1::GFP in the worm head by large-particle flow cytometry. Plot shows partial profile of head fluorescence from individual animals (gray circles). Mean ± SEM, ***p = 0.0002 by two-sided unpaired t-test, n = 201 animals for empty vector (EV), n = 200 animals for *kgb-1* RNAi over two independent trials. (**e**) Fluorescence micrographs of VHP-1::GFP expression in the head of day 2 adult animals on empty vector (EV), *kgb-1* RNAi, or *vhp-1* RNAi. Differential interference contrast (DIC). Reference Supplementary Fig. 6d. Scale = 50 µm. (**f**) Western blot of worms expressing VHP-1::GFP with different dilutions of *vhp-1* RNAi combined with the other indicated RNAi. (Top) anti-GFP, (bottom) anti-tubulin. (**g,h**) qPCR of *vhp-1* and *lys-3* transcript with injury (blue) and without injury (black) on the respective RNAi. Mean ± SEM, n = 3 biological replicates. See Supplementary Data 5 for statistics.

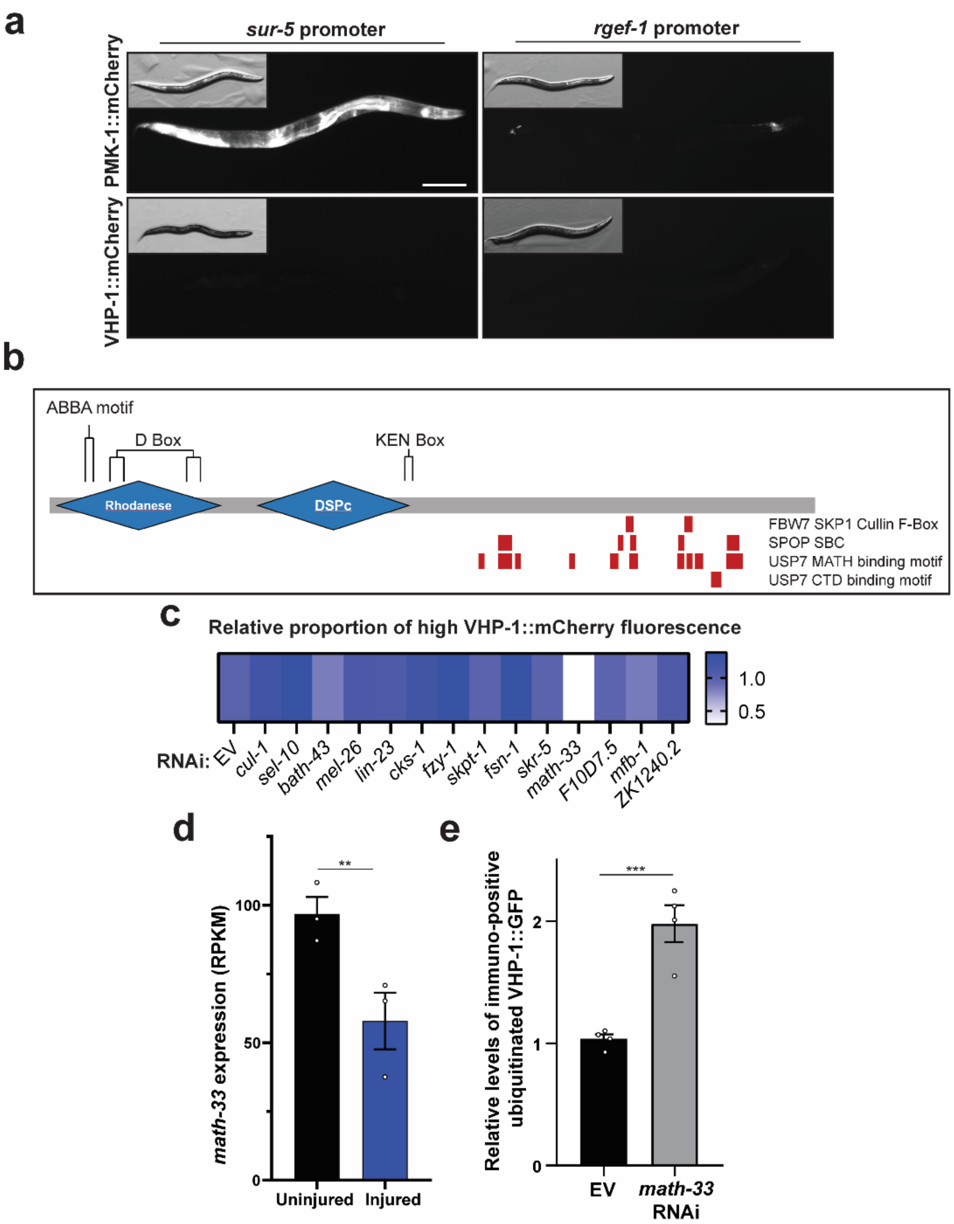

Supplementary Figure 7. VHP-1 steady state levels are affected by the proteasome or related components. (**a**) Fluorescence micrographs depict worms expressing neuronal (PMD101) or all-tissue (PMD106) PMK-1::mCherry as well as worms expressing neuronal (PMD107) or all-tissue (PMD117) VHP-1::mCherry. Insert, phase contrast. n = 4 independent replicates. Scale = 200 µm. (**b**) Model of full-length VHP-1 with predicated degrons (APC/C degron repository) and homologous short linear motifs for ubiquitin machinery (red, ELM server). (**c**) Heat map depicts results from reverse genetic screen by RNAi knockdown of genes representing ubiquitin-proteasome pathway components predicted to interact with VHP-1 by short-linear motif analysis (ELM server). EV, empty vector. Scale, blue and white intensity correspond to relative proportion of worms expressing VHP-1::mCherry in all tissues (PMD117) that fall with a high-fluorescence gate by large-particle flow cytometry. (**d**) RNAseq of *math-33* mRNA expression with injury (blue) or without injury (black). RPKM, reads per kilobase million. Mean ± SEM, *p = 0.0087 by Baggerly's test, n = 3 biological replicates. (**e**) Quantification of high-molecular weight, immuno-positive ubiquitinated VHP-1::GFP from GFP immunoprecipitations/western blots in Fig. 7g relative to empty vector (EV). Mean ± SEM, ***p = 0.009 by two-sided unpaired t-test, n = 4 biological replicates.

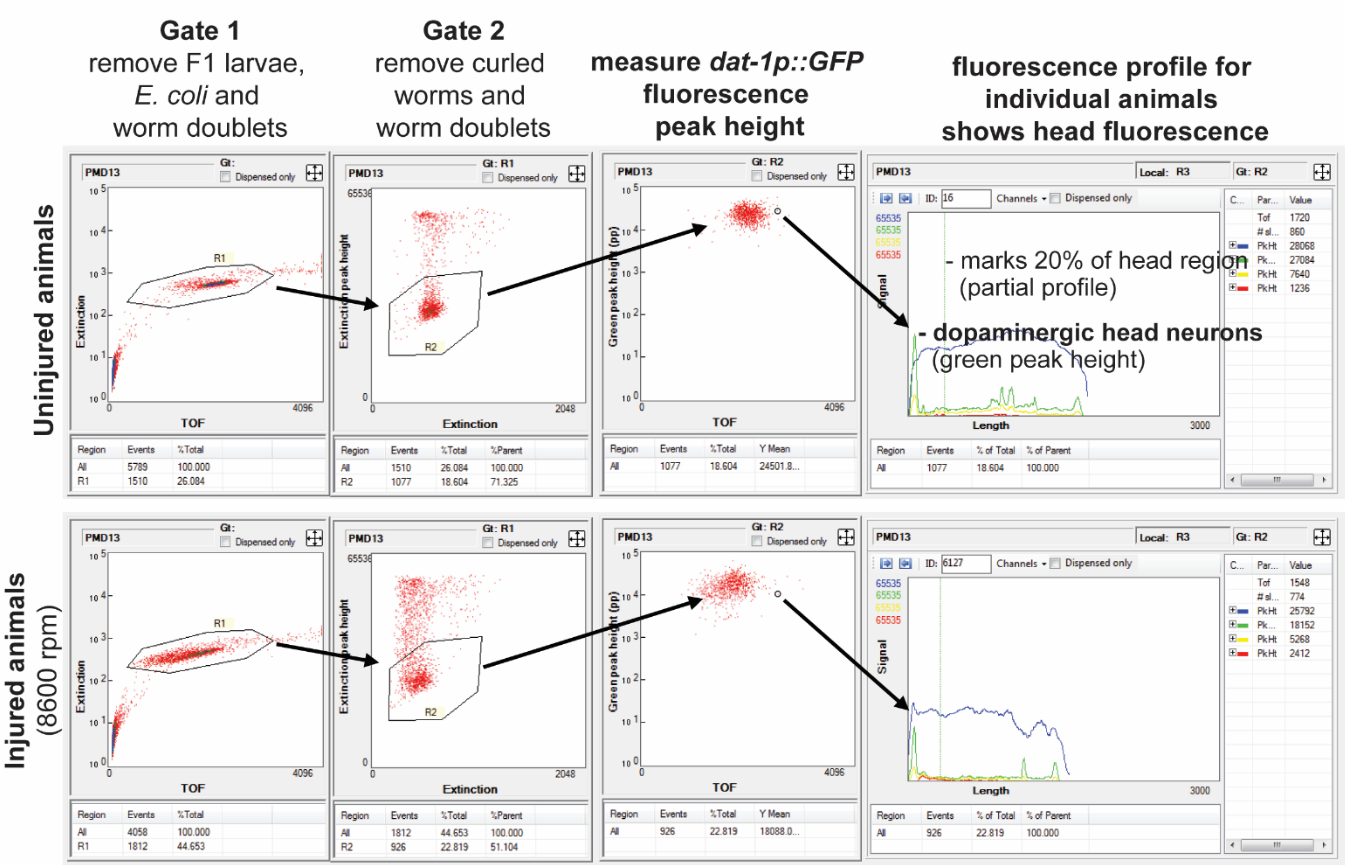

Supplementary Figure 8. Gating strategies for large-particle flow cytometry of *C. elegans*. Gating strategies used to analyze *dat-1p::GFP* fluorescence within head neurons. Shown are two representative gating methods which compare Day 2 uninjured animals (top) to day 2 adult worms subject to a single injury (bottom). Individual animal fluorescence profiles, right.

Figure	Strain, Treatment	Median Lifespan ± SD	Observed/total	% Lifespan increase	p-value log-rank (Mendel-Cox)	Compared to
2C	CF512, Uninjured	19.0+1.0	280/307			
2C	CF512, Day 1 injury	16.7+1.2	274/325	-12.3	<0.0001	Uninjured
2C	CF512, Day 4 injury	13.3+1.2	272/332	-29.8	<0.0001	Day 1 injury

Supplementary Table 1. Worm aging analysis after blunt force trauma.

RNAi Conditions	Retention Index	STD	n-value	p-value	Statistical Test
**MAPK**					
Empty Vector (EV)	0	2	3632 (4 trials)		
kgb-1 + EV	-0.03	1.609	1474 (2 trials)	>0.9999	one-way ANOVA with Tukey test
pmk-1 + EV	0.332	2.656	1372 (2 trials)	0.0026	one-way ANOVA with Tukey test
pmk-3 + EV	-0.145	2.337	2751 (3 trials)	0.5218	one-way ANOVA with Tukey test
vhp-1 + EV	0.764	3.31	8946 (3 trials)		
kgb-1 + vhp-1	0.665	2.038	1525 (3 trials)	0.9663	one-way ANOVA with Tukey test
pmk-1 + vhp-1	0.804	2.369	5168 (3 trials)	0.9991	one-way ANOVA with Tukey test
pmk-3 + vhp-1	0.652	2.734	5630 (4 trials)	0.2941	one-way ANOVA with Tukey test
jnk-1 + vhp-1	0.947	0.903	2766 (2 trials)	0.0372	one-way ANOVA with Tukey test
1/3 vhp-1 + EV	0.676	1.528	1044 (2 trials)		
kgb-1 + pmk-1 + vhp-1	0.577	1.636	2506 (2 trials)	0.9966	one-way ANOVA with Tukey test
kgb-1 + pmk-3 + vhp-1	0.488	1.94	3116 (2 trials)	0.6569	one-way ANOVA with Tukey test
pmk-1 + pml-3 + vhp-1	0.776	1.314	478 (1 trial)	>0.9999	one-way ANOVA with Tukey test
kgb-1 + jnk-1 + vhp-1	0.51	1.134	1959 (2 trials)	0.956	one-way ANOVA with Tukey test
**MAP2K**					
Empty Vector (EV)	0	2	2569 (3 trials)		
mek-1 + EV	0.073	1.157	1350 (2 trials)	0.965	one-way ANOVA with Tukey test
mkk-4 + EV	0.274	1.286	1273 (2 trials)	0.0022	one-way ANOVA with Tukey test
jkk-1 + EV	-0.124	1.133	1302 (2 trials)	0.6276	one-way ANOVA with Tukey test
vhp-1 + EV	0.951	2.575	4683 (3 trials)		
mek-1 + vhp-1	0.845	2.377	3802 (3 trials)	0.2556	one-way ANOVA with Tukey test
mkk-4 + vhp-1	0.919	1.832	1906 (2 trials)	0.9993	one-way ANOVA with Tukey test
jkk-1 + vhp-1	1	2	2350 (2 trials)	0.9798	one-way ANOVA with Tukey test
**MAP3K**					
Empty Vector (EV)	0	2	4834 (3 trials)		
mlk-1 + EV	-0.067	1.214	2082 (2 trials)	0.9784	one-way ANOVA with Tukey test
dlk-1 + EV	0.203	1.222	2392 (2 trials)	0.002	one-way ANOVA with Tukey test
nsy-1 + EV	0.076	2.157	4736 (4 trials)	0.7636	one-way ANOVA with Tukey test
vhp-1 + EV	0.9	2.247	5993 (4 trials)		
mlk-1 + vhp-1	0.785	2.566	10035 (3 trials)	0.0173	one-way ANOVA with Tukey test
dlk-1 + vhp-1	0.909	1.595	3661 (2 trials)	>0.9999	one-way ANOVA with Tukey test
nsy-1 + vhp-1	0.977	1.698	2503 (2 trials)	0.8878	one-way ANOVA with Tukey test
1/3 vhp-1 + EV	0.832	1.571	4006 (2 trials)		
mlk-1 + dlk-1 + vhp-1	0.184	1.324	2890 (2 trials)	<0.0001	one-way ANOVA with Tukey test
mlk-1 + nsy-1 + vhp-1	1.247	1.189	1228 (2 trials)	<0.0001	one-way ANOVA with Tukey test
dlk-1 + nsy-1 + vhp-1	1.138	1.126	2338 (2 trials)	<0.0001	one-way ANOVA with Tukey test

Supplementary Table 2: Impact of MAPK signaling pathway components on dopaminergic GFP retention after trauma.

Application	Primer Name	Nucleotide Sequence
Genotyping	*sid-1(pk3321)* FWD	ACTTCCGACGGTTTATCCGA
Genotyping	*sid-1(pk3321)* REV	GCACGGTTACGATCAGGTTT
Cloning	*vhp-1* 3'UTR FWD	CGGGGTACCTTTTGTGAACATCATTCTCTAGTCC
Cloning	*vhp-1* 3'UTR REV	CCCAAGCTTGGAAGGGGGTCAGGGCTCAAAATC
qPCR	*vhp-1* FWD	ATGACTTGCCCCAAATCCGT
qPCR	*vhp-1* REV	AATAGCCAGTGTGGGGCTTC
qPCR	*lys-3* FWD	TTGCACCAATGGCTGTGAGA
qPCR	*lys-3* REV	TCCAGCCTCCTGTGATTTCC
qPCR	*math-33* FWD	CACGGACTACAAAGAGCGGA
qPCR	*math-33* REV	TCTTCGGTGAGCATTGGTCC
qPCR	*tba-1* FWD	TCCACTGATCTCTGCTGACAA
qPCR	*tba-1* REV	TGGATCGCACTTCACCATT
qPCR	Y45F10D.4 FWD	TCTTCCCTGGCAACCGAATG
qPCR	Y45F10D.4 REV	CTTGGGCGAGCATTGAACAG

Supplementary Table 3. Description of oligonucleotides.
