# Supplementary material for: Trauma-induced regulation of VHP-1 modulates the cellular response to mechanical stress
Source: Nat Commun. 2021 Mar 5;12:1484. doi: 10.1038/s41467-021-21611-8 (PMC7935884; doi:10.1038/s41467-021-21611-8)
Supplement: Supplementary file 2 — Description of Additional Supplementary Files [file 41467_2021_21611_MOESM2_ESM.docx]

**Description of Additional Supplementary Files**

**Supplementary Data 1: Protein classes corresponding to transcripts activated by mechanical stress.**

**Supplementary Data 2: Mechanical stress responsive transcripts within the nervous system.**

**Supplementary Data 3: Validation of stress responsive RNAi.** Availability and sequence validation of HT115 *E. coli* which express the respective RNAi constructs from either the Vidal or Ahringer library.

**Supplementary Data 4: Mechanical stress response screen.** Results from the blunt force trauma screen in Figure 3e. Transcriptional fold change as well as corresponding mammalian genes with homology or sequence similarity are provided.

**Supplementary Data 5: Additional statistics for Figures and Supplementary Figures.**

**Supplementary Data 6: Cross species comparison of transcriptional changes after trauma in worm and mouse.** Shown are mammalian genes with homology or sequence similarity in relation to stress responsive worm genes. Mouse transcriptional changes analyzed 4 hours after controlled cortical impact (CCI) compared to sham surgery. Reference Supplementary Figure 3d.
