# Supplementary material for: Trauma-induced regulation of VHP-1 modulates the cellular response to mechanical stress
Source: Nat Commun. 2021 Mar 5;12:1484. doi: 10.1038/s41467-021-21611-8 (PMC7935884; doi:10.1038/s41467-021-21611-8)
Supplement: Supplementary file 9 — Reporting Summary [file 41467_2021_21611_MOESM9_ESM.pdf]

nature research

Corresponding author(s): Peter M. Douglas

Last updated by author(s): Jan 5, 2021

# Reporting Summary

Nature Research wishes to improve the reproducibility of the work that we publish. This form provides structure for consistency and transparency in reporting. For further information on Nature Research policies, see our Editorial Policies and the Editorial Policy Checklist.

## Statistics

For all statistical analyses, confirm that the following items are present in the figure legend, table legend, main text, or Methods section.

| n/a | Confirmed | |
|---|---|---|
| ☐ | ☒ | The exact sample size (*n*) for each experimental group/condition, given as a discrete number and unit of measurement |
| ☐ | ☒ | A statement on whether measurements were taken from distinct samples or whether the same sample was measured repeatedly |
| ☐ | ☒ | The statistical test(s) used AND whether they are one- or two-sided<br>*Only common tests should be described solely by name; describe more complex techniques in the Methods section.* |
| ☐ | ☒ | A description of all covariates tested |
| ☐ | ☒ | A description of any assumptions or corrections, such as tests of normality and adjustment for multiple comparisons |
| ☐ | ☒ | A full description of the statistical parameters including central tendency (e.g. means) or other basic estimates (e.g. regression coefficient) AND variation (e.g. standard deviation) or associated estimates of uncertainty (e.g. confidence intervals) |
| ☐ | ☒ | For null hypothesis testing, the test statistic (e.g. *F*, *t*, *r*) with confidence intervals, effect sizes, degrees of freedom and *P* value noted<br>*Give P values as exact values whenever suitable.* |
| ☒ | ☐ | For Bayesian analysis, information on the choice of priors and Markov chain Monte Carlo settings |
| ☒ | ☐ | For hierarchical and complex designs, identification of the appropriate level for tests and full reporting of outcomes |
| ☒ | ☐ | Estimates of effect sizes (e.g. Cohen's *d*, Pearson's *r*), indicating how they were calculated |

*Our web collection on statistics for biologists contains articles on many of the points above.*

## Software and code

Policy information about availability of computer code

| | |
|---|---|
| Data collection | Software used for data collection include FlowPilot (ver. 1.6.18, Union Biometrica), Image Studio (ver. 5, LI-COR), ImageJ (ver. 1.52n), Leica Application Suite X (LAS X ver. 3.5.5.19976) |
| Data analysis | Software for data analysis include FlowPilot (ver. 1.6.18), Excel (ver. 16, Microsoft), CLC Genomics (ver. 9.5, CLC bio), ImageJ (ver. 1.5), Image Studio Lite (ver. 5.2, LI-COR), Prism (ver. 8.4, GraphPad), WormBase Enrichment Analysis (ver. WS278) |

For manuscripts utilizing custom algorithms or software that are central to the research but not yet described in published literature, software must be made available to editors and reviewers. We strongly encourage code deposition in a community repository (e.g. GitHub). See the Nature Research guidelines for submitting code & software for further information.

## Data

Policy information about availability of data

All manuscripts must include a data availability statement. This statement should provide the following information, where applicable:

- Accession codes, unique identifiers, or web links for publicly available datasets
- A list of figures that have associated raw data
- A description of any restrictions on data availability

All data generated and analyzed during this study are included in this article and its Supplementary Information, and are also available from the authors upon reasonable request. Transcriptomic data files that support the findings of this study on mechanical stress in C. elegans have been deposited in the NCBI Gene Expression Omnibus (GEO). Illumina HiSeq 2500 datasets were assigned an accession code of GSE148337 and an identifier of 200148337 (https://www.ncbi.nlm.nih.gov/geo/query/acc.cgi?acc=GSE148337). Affimetrix C. elegans Genome microarrays were assigned an accession code of GSE148325 and an identifier of 200148325 (https://www.ncbi.nlm.nih.gov/geo/query/acc.cgi?acc=GSE148325).

# Field-specific reporting

Please select the one below that is the best fit for your research. If you are not sure, read the appropriate sections before making your selection.

- [x] Life sciences
- [ ] Behavioural & social sciences
- [ ] Ecological, evolutionary & environmental sciences

For a reference copy of the document with all sections, see nature.com/documents/nr-reporting-summary-flat.pdf

# Life sciences study design

All studies must disclose on these points even when the disclosure is negative.

| | |
|---|---|
| Sample size | When sample size calculation was relevant to perform, desired power was set to 0.80 and alpha to 0.05. Sample size calculation was performed utilizing standard equations for either dichotomous or continuous variables, as appropriate. Large particle flow cytometry experiments: sampling a smaller portion of the larger animal populations was not necessary because large particle flow cytometry enabled rapid collection and analysis for the entire population of worms. qPCR: Desired at minimum to detect a 2-fold change. Preliminary experiments revealed that the coefficient of variation (CV) for vhp-1 expression data was 0.15. With the above power and significance, we obtained a minimum sample size of approximately 3. Nose-touch reversals: Desired at minimum to detect a relative difference of 20% in response rate. Common CV from first experiment was 0.16, indicating scoring at least 11 worms per condition could provide a power of 0.80 or greater. Movement defects: Initial experiment revealed normal movement in approximately 74% scored uninjured worms and 40% mildly injured worms, indicating sample size of at least 32 worms per group for adequate statistical power. Paralysis: In scoring moderate vs mild injury, initial experiments indicated paralysis incidence of 84.9% and 10.8%, respectively. For a power of 0.8, minimum group sample size was calculated to be 6. Chemotaxis: Initial experiment suggested 90% uninjured worms were butanol-responsive while only 56% of injured worms were. For power of 0.8, a minimum of 26 worms would need to be scored per group. Microscopy: To evaluate neuron degeneration, initial experiments revealed uninjured and injured worms displayed 0% and 45.3% unhealthy neurons, respectively. To detect a difference of at least 20% with power of 0.8, a minimum of 34 neurons are needed per group. |
| Data exclusions | Two of the RNAseq samples (vhp-1 with and without injury) were excluded in recommendation by Novogene as the samples received a hold during quality control testing. |
| Replication | Data were examined for effect size, distribution, and normality. Individual controls were utilized within each experiment such that effect size was controlled from experiment to experiment. Within the figures, figure legends and supplementary data, n values were reported and often total worm number as well as the number of trials performed to ensure reproducibility. |
| Randomization | Allocation of plates containing worms was random. Although, this may not be relevant to our study due to the inherent random component when utilizing thousands of animals per condition that are not observed before allocation. For imaging, worms were randomly picked from each population. Mice were housed together regardless of genotype. For experimentation, mice were grouped by sex and age for control. |
| Blinding | For most worm experiments entailing the automated large-particle flow cytometer, blinding was less relevant as 1000s of worms across several conditions (in some cases 50-60 conditions) were measured using automated means from 96-well plates. In experiments involving several of the gene products, which are the focus of this study, RNAi treatments or mutations produced noticeable anatomical or behavioral phenotypes that divulged the nature of the respective conditions and often negated the blinding process. Nonetheless, blinding for worm experiments (including trauma titrations as well as behavioral and motility assays) was performed by assigning culturing plates randomly selected letters or numbers. All image collection and analysis was performed in a similar manner. Mouse experiments were partially blinded where mouse ID was known, but genotype was hidden. |

# Reporting for specific materials, systems and methods

We require information from authors about some types of materials, experimental systems and methods used in many studies. Here, indicate whether each material, system or method listed is relevant to your study. If you are not sure if a list item applies to your research, read the appropriate section before selecting a response.

## Materials & experimental systems

| n/a | Involved in the study | |
|---|---|---|
| ☐ | ☒ | Antibodies |
| ☒ | ☐ | Eukaryotic cell lines |
| ☒ | ☐ | Palaeontology and archaeology |
| ☐ | ☒ | Animals and other organisms |
| ☒ | ☐ | Human research participants |
| ☒ | ☐ | Clinical data |
| ☒ | ☐ | Dual use research of concern |

## Methods

| n/a | Involved in the study | |
|---|---|---|
| ☒ | ☐ | ChIP-seq |
| ☐ | ☒ | Flow cytometry |
| ☒ | ☐ | MRI-based neuroimaging |

## Antibodies

**Antibodies used**

Tubulin, GFP, DUSP16, KGB-1, p38, ERK, phospho-ERK, phospho-KGB-1 and phospho-p38, ubiquitin.
Details: Mouse anti-aTubulin (Cat. No. T6074; Sigma), mouse anti-GFP (Sigma, Cat. No. 11814460001), rabbit anti-GFP (Thermo Fisher, Cat. No. A6455), rabbit anti-DUSP16 (Cell Signaling, Cat. No. 5523S), rabbit anti-KGB-1 (kind gift from the Matsumoto lab), rabbit phospho-KGB-1 (kind gift from the Matsumoto lab), rabbit anti-phospho-p38 (Cell Signaling, Cat. No. 9211S), rabbit anti-p38 (Cell Signaling, Cat. No. 9212S), rabbit phospho-ERK1/2 (Cell Signaling, Cat. No. 9101S), rabbit ERK1/2 (Cell Signaling, Cat. No. 9102S), rabbit anti-ubiquitin (Abcam, Cat. No. ab19247).

**Validation**

All commercially validated antibodies were further validated in most all cases through knockdown of the the respective protein in worms. The commercial DUSP16 antibody produced immunoreactive species at the predicted molecular weight of the DUSP16 protein in mouse brain homogenates. We further validated this DUSP16 antibody via transient transfections of a HA epitope tagged DUSP16 plasmid within both HEK293 and NIH3T3 cells. Control transfections included the HA plasmid alone. Only with the DUSP16-HA transfection did we observe overlapping HA and DUSP16 immunoreactive bands at the predicted molecular weight.

## Animals and other organisms

Policy information about studies involving animals; ARRIVE guidelines recommended for reporting animal research

**Laboratory animals**

Mus musculus: strain B6D2Fl/J included 10-12 week old males group housed on 12 hour light/dark cycles at 22 degrees Celsius and 46% humidity. Caenorhabditis elegans: strains N2, CF512, AGD1651, KM20, KB3, JT366, RB2194, LC108, BZ555, TU3401, CZ1632, PMD13, PMD14, PMD60, PMD62, PMD63, PMDl0l, PMD106, PMD107, PMD112, PMD117, PMD152, PMD153, PMD173.

**Wild animals**

The study did not involve wild animals.

**Field-collected samples**

The study did not involve samples collected from the field.

**Ethics oversight**

IACUC approval for closed head traumatic brain injury is APN 2017102030 and approval for characterizing the role of the Dual Specific Phosphatase 16 in the mouse brain is APN 2019102807.

Note that full information on the approval of the study protocol must also be provided in the manuscript.

## Flow Cytometry

### Plots

Confirm that:

- [ ] The axis labels state the marker and fluorochrome used (e.g. CD4-FITC).
- [x] The axis scales are clearly visible. Include numbers along axes only for bottom left plot of group (a 'group' is an analysis of identical markers).
- [ ] All plots are contour plots with outliers or pseudocolor plots.
- [x] A numerical value for number of cells or percentage (with statistics) is provided.

### Methodology

**Sample preparation**

This analysis does not pertain to cells but rather large-particle flow cytometry of whole animals. C. elegans were grown on NGM agarose plates to various ages and on various RNAi treatments. Worms were collected by washing them off the plate with M9 liquid media, washed 2 times with 1000 x g centrifugation, concentrated in 100 microliters of M9 media, and transferred to 96-well plates for automated large-particle flow cytometry.

**Instrument**

Union Biometrica COPAS FP-250 with LPS

**Software**

Flowpilot (ver. 1.6.18)

**Cell population abundance**

Worm populations vary from 100s to several 1000s of animals per trial.

**Gating strategy**

Gate 1- First, animals were gated based on "extinction" to "time of flight" to select for worms size (adults) and remove bacteria, debris, eggs and F1 larvae. Gate 2- next, animals were gated based on "extinction peak height" to "extinction" to remove curled up worms or doublets going through the flow cell simultaneously. Gate 3- at this point, gating strategies varied depending on the experiment. For all experiments looking at dopaminergic neurodegeneration, "green peak height" directly measured fluorescence in the animal's head CEP and ADE neurons and no further gating was needed. For translational GFP fluorescence reporting transgenic animals, DiI was supplemented prior to large particle flow cytometry to mark the head region and enable animal orientation. Head fluorescence within the front 20% of each individual animal was measured to give neuronal fluorescence quantification.

- [x] Tick this box to confirm that a figure exemplifying the gating strategy is provided in the Supplementary Information.
